# Supplementary material for: Rat BodyMap transcriptomes reveal unique circular RNA features across tissue types and developmental stages
Source: RNA. 2018 Nov;24(11):1443–56. doi: 10.1261/rna.067132.118 (PMC6191709; doi:10.1261/rna.067132.118)
Supplement: Supplemental Material [file supp_24_11_1443__index.html]

Rat BodyMap transcriptomes reveal unique circular RNA features across tissue types and developmental stages — Supplemental Material 

# Rat BodyMap transcriptomes reveal unique circular RNA features across tissue types and developmental stages

## Supplemental Material

- Supplemental\_Legends.docx
- Supplemental\_Tables.pdf
- Supplemental\_Figures.pdf
