# Supplementary material for: Rat BodyMap transcriptomes reveal unique circular RNA features across tissue types and developmental stages
Source: RNA. 2018 Nov;24(11):1443–56. doi: 10.1261/rna.067132.118 (PMC6191709; doi:10.1261/rna.067132.118)
Supplement: Supplemental Material [file supp_067132.118_Supplemental_Tables.pdf]

Supplementary Table S1. The host genes of the age-dependent circRNAs in brain

Ensembl ID	Gene name	*ρ*
ENSRNOG00000000942	PAN3 poly(A) specific ribonuclease subunit(Pan3)	0.845
ENSRNOG00000001283	anaphase promoting complex subunit 7(Anapc7)	0.760
ENSRNOG00000002075	CCR4-NOT transcription complex, subunit 6-like(Cnot6l)	0.753
ENSRNOG00000002214	kelch-like family member 8(Klhl8)	0.823
ENSRNOG00000002983	nuclear factor 1 X(Nf1x)	0.817
ENSRNOG00000003399	heterogeneous nuclear ribonucleoprotein H1(Hnrnph1)	0.736
ENSRNOG00000003882	centrosomal protein 350(Cep350)	0.884
ENSRNOG00000004050	SNF related kinase(Snrk)	0.842
ENSRNOG00000004736	Ral GEF with PH domain and SH3 binding motif 2(Ralgps2)	0.736
ENSRNOG00000004823	dedicator of cytokinesis 4(Dock4)	0.718
ENSRNOG00000005291	solute carrier family 38, member 1(Slc38a1)	0.708
ENSRNOG00000005502	ankyrin repeat and IBR domain containing 1(Ankib1)	0.745
ENSRNOG00000005624	zinc finger protein X-linked(Zfx)	0.811
ENSRNOG00000006841	anoctamin 4(Ano4)	0.763
ENSRNOG00000009974	coenzyme Q3 methyltransferase(Coq3)	0.734
ENSRNOG00000010150	spermatid perinuclear RNA binding protein(Strbp)	0.745
ENSRNOG00000010489	sterile alpha motif domain containing 4A(Samd4a)	0.727
ENSRNOG00000010637	TBC1 domain family, member 5(Tbc1d5)	0.817
ENSRNOG00000010815	E74-like factor 2(Elf2)	0.827
ENSRNOG00000011000	regulating synaptic membrane exocytosis 1(Rims1)	0.720
ENSRNOG00000011063	DENN domain containing 1B(Dennd1b)	0.745
ENSRNOG00000011278	serine/threonine kinase 3(Stk3)	0.839
ENSRNOG00000011358	homeodomain interacting protein kinase 3(Hipk3)	0.784
ENSRNOG00000011420	myotubularin related protein 7(Mtmr7)	0.751
ENSRNOG00000011914	dystrobrevin, beta(Dtnb)	0.811
ENSRNOG00000012653	protein phosphatase 1, regulatory subunit 13B(Ppp1r13b)	0.815
ENSRNOG00000012899	RB binding protein 8, endonuclease(Rbbp8)	0.729
ENSRNOG00000014531	HBS1-like translational GTPase(Hbs1l)	0.704
ENSRNOG00000015189	Bardet-Biedl syndrome 9(Bbs9)	0.724
ENSRNOG00000016206	UV radiation resistance associated(Uvrag)	0.866
ENSRNOG00000016213	single-stranded DNA binding protein 2(Ssbp2)	0.757
ENSRNOG00000017940	arginine-glutamic acid dipeptide repeats(Rere)	0.811
ENSRNOG00000018674	neurotrophic receptor tyrosine kinase 3(Ntrk3)	0.802
ENSRNOG00000019476	ArfGAP with GTPase domain, ankyrin repeat and PH domain 1(Agap1)	0.733
ENSRNOG00000020386	ASH1 like histone lysine methyltransferase(Ash1l)	0.760
ENSRNOG00000020689	cytoplasmic polyadenylation element binding protein 3(Cpeb3)	0.730
ENSRNOG00000020769	CREB3 regulatory factor(Crebrf)	0.860
ENSRNOG00000021468	glutamate metabotropic receptor 8(Grm8)	0.821
ENSRNOG00000021569	T-cell lymphoma invasion and metastasis 1(Tiam1)	0.758
ENSRNOG00000021581	Rap guanine nucleotide exchange factor 2(Rapgef2)	0.851

Ensembl ID	Gene name	ρ
ENSRNOG00000023679	PR/SET domain 5(Prdm5)	0.799
ENSRNOG00000024808	serine threonine kinase 39(Stk39)	0.790
ENSRNOG00000024870	ankyrin repeat and sterile alpha motif domain containing 1B(Anks1b)	0.714
ENSRNOG00000026942	similar to KIAA2026 protein(RGD1311595)	0.796
ENSRNOG00000030213	vacuolar protein sorting 13C(Vps13c)	0.726
ENSRNOG00000032215	chromodomain Y-like(Cdyl)	0.839
ENSRNOG00000045605	UDP-glucuronate decarboxylase 1(Uxs1)	0.739
ENSRNOG00000046139	synaptotagmin 14(Syt14)	0.739
ENSRNOG00000051204	dopey family member 2(Dopey2)	0.832
ENSRNOG00000052721	lysine (K)-specific demethylase 6A(NEWGENE_1565481)	0.729
ENSRNOG00000054514	muskelin 1(Mkln1)	0.754
ENSRNOG00000057042	propionyl-CoA carboxylase alpha subunit(Pcca)	0.754
ENSRNOG00000060367	regulatory factor X, 7(Rfx7)	0.745

Note – The ρ values were calculated with *Spearman*'s rank correlation test. Only the circRNAs with $\rho > 0.7$ were listed.

Supplementary Table S2. The host genes of the age-dependent circRNAs in testes

Age	Ensembl ID	Gene name	*ρ*
2- to 21-week	ENSRNOG00000000262	zinc finger protein 821(Zfp821)	0.631
2- to 21-week	ENSRNOG00000000327	HECT domain and ankyrin repeat containing, E3 ubiquitin protein ligase 1(Hace1)	0.674
2- to 21-week	ENSRNOG00000000542	dynein, axonemal, heavy chain 8(Dnah8)	0.824
2- to 21-week	ENSRNOG00000000802	TBC1 domain family, member 32(Tbc1d32)	0.798
2- to 21-week	ENSRNOG00000001971	BBX, HMG-box containing(Bbx)	0.622
2- to 21-week	ENSRNOG00000002142	CDP-diacylglycerol synthase 1(Cds1)	0.725
2- to 21-week	ENSRNOG00000002204	coiled-coil domain containing 158(Ccdc158)	0.699
2- to 21-week	ENSRNOG00000002504	spermatogenesis associated 17(Spata17)	0.651
2- to 21-week	ENSRNOG00000002512	G patch domain containing 2(Gpatch2)	0.710
2- to 21-week	ENSRNOG00000002516	myotubularin 1(Mtm1)	0.963
2- to 21-week	ENSRNOG00000002544	kinesin-associated protein 3(Kifap3)	0.773
2- to 21-week	ENSRNOG00000002736	RAB GTPase activating protein 1-like(Rabgap1l)	0.797
2- to 21-week	ENSRNOG00000002755	platelet-activating factor acetylhydrolase 1b, regulatory subunit 1(Pafah1b1)	0.655
2- to 21-week	ENSRNOG00000002882	zinc finger CCHC-type containing 2(Zcchc2)	0.631
2- to 21-week	ENSRNOG00000003078	DDB1 and CUL4 associated factor 6(Dcaf6)	0.621
2- to 21-week	ENSRNOG00000003123	replication protein A1(Rpa1)	0.699
2- to 21-week	ENSRNOG00000003186	ankyrin repeat and sterile alpha motif domain containing 3(Anks3)	0.694
2- to 21-week	ENSRNOG00000003258	cell division cycle 73(Cdc73)	0.783
2- to 21-week	ENSRNOG00000003273	spermatogenesis associated 20(Spata20)	0.889
2- to 21-week	ENSRNOG00000003695	MGAT4 family, member D(Mgat4d)	0.726
2- to 21-week	ENSRNOG00000003908	centrosomal protein 128(Cep128)	0.769
2- to 21-week	ENSRNOG00000003955	spermatogenesis associated 7(Spata7)	0.699
2- to 21-week	ENSRNOG00000003991	ankyrin and armadillo repeat containing(Ankar)	0.873
2- to 21-week	ENSRNOG00000004076	PMS1 homolog 1, mismatch repair system component(Pms1)	0.616
2- to 21-week	ENSRNOG00000004201	regulating synaptic membrane exocytosis 2(Rims2)	0.903
2- to 21-week	ENSRNOG00000004290	growth factor receptor bound protein 10(Grb10)	0.694
2- to 21-week	ENSRNOG00000004448	acyl-CoA synthetase short-chain family member 3(Acss3)	0.699
2- to 21-week	ENSRNOG00000004456	clathrin heavy chain linker domain containing 1(Clhc1)	0.686
2- to 21-week	ENSRNOG00000004564	leucine-rich repeats and IQ motif containing 1(Lrriq1)	0.783
2- to 21-week	ENSRNOG00000005042	RAN binding protein 17(Ranbp17)	0.798
2- to 21-week	ENSRNOG00000005101	cutaneous T-cell lymphoma-associated antigen 5 homolog(LOC100912115)	0.686
2- to 21-week	ENSRNOG00000005105	Sad1 and UNC84 domain containing 3(Sun3)	0.651
2- to 21-week	ENSRNOG00000005233	leucine rich repeat containing 6(Lrrc6)	0.694
2- to 21-week	ENSRNOG00000005883	NIMA-related kinase 10(Nek10)	0.686
2- to 21-week	ENSRNOG00000005907	RAD18 E3 ubiquitin protein ligase(Rad18)	0.798
2- to 21-week	ENSRNOG00000005916	protein phosphatase, Mg2+/Mn2+ dependent, 1A(Ppm1a)	0.710
2- to 21-week	ENSRNOG00000005934	muscular LMNA-interacting protein(Mlip)	0.857
2- to 21-week	ENSRNOG00000006114	cilia and flagella associated protein 69(Cfap69)	0.626
2- to 21-week	ENSRNOG00000006248	tripartite motif-containing 37(Trim37)	0.873

Age	Ensembl ID	Gene name	ρ
2- to 21-week	ENSRNOG00000006304	MDM2 proto-oncogene(Mdm2)	0.679
2- to 21-week	ENSRNOG00000006340	mediator of cell motility 1(Memo1)	0.770
2- to 21-week	ENSRNOG00000006785	DDB1 and CUL4 associated factor 8(Dcaf8)	0.725
2- to 21-week	ENSRNOG00000006832	zinc finger, DHHC-type containing 5(Zdhhc5)	0.725
2- to 21-week	ENSRNOG00000007034	homeodomain interacting protein kinase 2(Hipk2)	0.699
2- to 21-week	ENSRNOG00000007127	Bardet-Biedl syndrome 5(Bbs5)	0.602
2- to 21-week	ENSRNOG00000007341	maestro heat-like repeat family member 8(Mroh8)	0.686
2- to 21-week	ENSRNOG00000007523	chaperonin containing TCP1 subunit 6B(Cct6b)	0.692
2- to 21-week	ENSRNOG00000007730	vomeronasal 2 receptor, 53(Vom2r53)	0.844
2- to 21-week	ENSRNOG00000008039	cullin 5(Cul5)	0.651
2- to 21-week	ENSRNOG00000008053	ATPase phospholipid transporting 8A2(Atp8a2)	0.920
2- to 21-week	ENSRNOG00000008393	Tax1 binding protein 1(Tax1bp1)	0.873
2- to 21-week	ENSRNOG00000008775	leucine-rich repeats and guanylate kinase domain containing(Lrguk)	0.736
2- to 21-week	ENSRNOG00000008859	TRAF family member-associated NFKB activator(Tank)	0.834
2- to 21-week	ENSRNOG00000009208	progesterone immunomodulatory binding factor 1(Pibf1)	0.791
2- to 21-week	ENSRNOG00000009264	ELKS/RAB6-interacting/CAST family member 1(Erc1)	0.681
2- to 21-week	ENSRNOG00000009338	KRAS proto-oncogene, GTPase(Kras)	0.920
2- to 21-week	ENSRNOG00000009555	leucine-rich repeats and IQ motif containing 3(Lrriq3)	0.674
2- to 21-week	ENSRNOG00000009664	ankyrin repeat domain 42(Ankrd42)	0.873
2- to 21-week	ENSRNOG00000009696	MDM4, p53 regulator(Mdm4)	0.699
2- to 21-week	ENSRNOG00000009745	protein phosphatase 3 catalytic subunit gamma(Ppp3cc)	0.681
2- to 21-week	ENSRNOG00000009766	G protein-coupled receptor 180(Gpr180)	0.699
2- to 21-week	ENSRNOG00000009792	zinc finger, B-box domain containing(Zbbx)	0.757
2- to 21-week	ENSRNOG00000009808	intraflagellar transport 80(Ift80)	0.767
2- to 21-week	ENSRNOG00000009851	solute carrier family 38, member 9(Slc38a9)	0.889
2- to 21-week	ENSRNOG00000010024	cytochrome b5 reductase 4(Cyb5r4)	0.625
2- to 21-week	ENSRNOG00000010027	ATR serine/threonine kinase(Atr)	0.631
2- to 21-week	ENSRNOG00000010150	spermatid perinuclear RNA binding protein(Strbp)	0.650
2- to 21-week	ENSRNOG00000010155	pericentriolar material 1(Pcm1)	0.632
2- to 21-week	ENSRNOG00000010418	NIMA-related kinase 1(Nek1)	0.804
2- to 21-week	ENSRNOG00000010526	DENN domain containing 1A(Dennd1a)	0.828
2- to 21-week	ENSRNOG00000010536	nuclear receptor subfamily 2, group C, member 2(Nr2c2)	0.651
2- to 21-week	ENSRNOG00000010637	TBC1 domain family, member 5(Tbc1d5)	0.887
2- to 21-week	ENSRNOG00000010815	E74-like factor 2(Elf2)	0.744
2- to 21-week	ENSRNOG00000010819	heat shock protein 4-like(Hspa4l)	0.694
2- to 21-week	ENSRNOG00000010952	intraflagellar transport 122(Ift122)	0.699
2- to 21-week	ENSRNOG00000011000	regulating synaptic membrane exocytosis 1(Rims1)	0.946
2- to 21-week	ENSRNOG00000011059	tau tubulin kinase 2(Ttbk2)	0.655
2- to 21-week	ENSRNOG00000011066	membrane associated ring-CH-type finger 6(March6)	0.791
2- to 21-week	ENSRNOG00000011094	EF-hand calcium binding domain 6(Efcab6)	0.686
2- to 21-week	ENSRNOG00000011376	basal body orientation factor 1(Bbof1)	0.616

Age	Ensembl ID	Gene name	ρ
2- to 21-week	ENSRNOG00000011382	WD repeat domain 33(Wdr33)	0.659
2- to 21-week	ENSRNOG00000011417	phosphodiesterase 3B(Pde3b)	0.710
2- to 21-week	ENSRNOG00000011603	calcium binding protein 39-like(Cab39l)	0.686
2- to 21-week	ENSRNOG00000011627	zinc finger RNA binding protein(Zfr)	0.644
2- to 21-week	ENSRNOG00000011669	glutathione S-transferase, C-terminal domain containing(Gstcd)	0.857
2- to 21-week	ENSRNOG00000011791	zinc finger AN1-type containing 4(Zfand4)	0.829
2- to 21-week	ENSRNOG00000011847	G protein-coupled receptor kinase 4(Grk4)	0.804
2- to 21-week	ENSRNOG00000012015	leucine rich repeat containing 49(Lrrc49)	0.616
2- to 21-week	ENSRNOG00000012055	mannosidase, alpha, class 2A, member 2(Man2a2)	0.722
2- to 21-week	ENSRNOG00000012536	sphingomyelin synthase 1(Sgms1)	0.744
2- to 21-week	ENSRNOG00000012637	amyotrophic lateral sclerosis 2 chromosome region, candidate 11(Als2cr11)	0.699
2- to 21-week	ENSRNOG00000012806	RB binding protein 6, ubiquitin ligase(Rbbp6)	0.631
2- to 21-week	ENSRNOG00000013293	methylcrotonoyl-CoA carboxylase 1(Mccc1)	0.699
2- to 21-week	ENSRNOG00000013318	coiled-coil domain containing 150(Ccdc150)	0.692
2- to 21-week	ENSRNOG00000013673	mutS homolog 3(Msh3)	0.705
2- to 21-week	ENSRNOG00000013876	mitochondrial intermediate peptidase(Mipep)	0.651
2- to 21-week	ENSRNOG00000013884	pleckstrin and Sec7 domain containing 3(Psd3)	0.798
2- to 21-week	ENSRNOG00000013962	membrane associated guanylate kinase, WW and PDZ domain containing 2(Magi2)	0.615
2- to 21-week	ENSRNOG00000014139	sodium channel and clathrin linker 1(Sclt1)	0.710
2- to 21-week	ENSRNOG00000014163	zinc finger protein 536(Zfp536)	0.699
2- to 21-week	ENSRNOG00000014276	phospholipase C, epsilon 1(Plce1)	0.686
2- to 21-week	ENSRNOG00000014486	regulatory factor X3(Rfx3)	0.828
2- to 21-week	ENSRNOG00000014576	dedicator of cyto-kinesis 3(Dock3)	0.887
2- to 21-week	ENSRNOG00000014584	outer dense fiber of sperm tails 2(Odf2)	0.659
2- to 21-week	ENSRNOG00000014590	serine/threonine kinase 33(Stk33)	0.699
2- to 21-week	ENSRNOG00000014775	nucleolar protein 4(Nol4)	0.844
2- to 21-week	ENSRNOG00000014875	ASH2 like histone lysine methyltransferase complex subunit(Ash2l)	0.705
2- to 21-week	ENSRNOG00000015162	lon peptidase 2, peroxisomal(Lonp2)	0.699
2- to 21-week	ENSRNOG00000015189	Bardet-Biedl syndrome 9(Bbs9)	0.917
2- to 21-week	ENSRNOG00000015357	BMP-binding endothelial regulator(Bmper)	0.647
2- to 21-week	ENSRNOG00000015519	carboxylesterase 1D(Ces1d)	0.946
2- to 21-week	ENSRNOG00000015734	ubiquitin protein ligase E3A(Ube3a)	0.681
2- to 21-week	ENSRNOG00000015898	FER tyrosine kinase(Fer)	0.821
2- to 21-week	ENSRNOG00000016183	intracisternal A particle-promoted polypeptide(Ipp)	0.844
2- to 21-week	ENSRNOG00000016360	G protein pathway suppressor 2(Gps2)	0.857
2- to 21-week	ENSRNOG00000016545	intraflagellar transport 140(Ift140)	0.699
2- to 21-week	ENSRNOG00000016620	casein kinase 1, gamma 1(Csnk1g1)	0.742
2- to 21-week	ENSRNOG00000016635	similar to K04F10.2(LOC361646)	0.699
2- to 21-week	ENSRNOG00000016703	general transcription factor 2A subunit 1 like(Gtf2a1l)	0.647
2- to 21-week	ENSRNOG00000016717	growth arrest-specific 2(Gas2)	0.707
2- to 21-week	ENSRNOG00000016910	Ral GEF with PH domain and SH3 binding motif 1(Ralgps1)	0.715

Age	Ensembl ID	Gene name	ρ
2- to 21-week	ENSRNOG00000016963	thyroid hormone receptor interactor 12(Trip12)	0.705
2- to 21-week	ENSRNOG00000016992	POTE ankyrin domain family, member C(Potec)	0.663
2- to 21-week	ENSRNOG00000017074	RAB28, member RAS oncogene family(Rab28)	0.692
2- to 21-week	ENSRNOG00000017178	Hydin, axonemal central pair apparatus protein(Hydin)	0.828
2- to 21-week	ENSRNOG00000017282	testis-specific kinase 2(Tesk2)	0.852
2- to 21-week	ENSRNOG00000017518	ADAM metallopeptidase domain 5(Adam5)	0.789
2- to 21-week	ENSRNOG00000017554	ADAM metallopeptidase domain 3A(Adam3a)	0.844
2- to 21-week	ENSRNOG00000017581	similar to 4833420G17Rik protein(RGD1306227)	0.616
2- to 21-week	ENSRNOG00000017608	C2 calcium-dependent domain containing 3(C2cd3)	0.679
2- to 21-week	ENSRNOG00000018345	ATP binding cassette subfamily E member 1(Abce1)	0.686
2- to 21-week	ENSRNOG00000018381	CDK5 regulatory subunit associated protein 1-like 1(Cdkal1)	0.674
2- to 21-week	ENSRNOG00000018387	WD repeat domain 7(Wdr7)	0.650
2- to 21-week	ENSRNOG00000018457	protein phosphatase 2 phosphatase activator(Ptpa)	0.699
2- to 21-week	ENSRNOG00000018529	casein kinase 1, gamma 2(Csnk1g2)	0.828
2- to 21-week	ENSRNOG00000018946	tripartite motif-containing 33(Trim33)	0.685
2- to 21-week	ENSRNOG00000019271	unc-51 like kinase 4(Ulk4)	0.828
2- to 21-week	ENSRNOG00000019272	G kinase anchoring protein 1(Gkap1)	0.726
2- to 21-week	ENSRNOG00000019752	solute carrier family 29 member 1(Slc29a1)	0.834
2- to 21-week	ENSRNOG00000019826	SIL1 nucleotide exchange factor(Sil1)	0.920
2- to 21-week	ENSRNOG00000020118	NME/NM23 family member 5(Nme5)	0.889
2- to 21-week	ENSRNOG00000020191	TBC1 domain family, member 17(Tbc1d17)	0.651
2- to 21-week	ENSRNOG00000020203	protein tyrosine phosphatase, non-receptor type 20(Ptpn20)	0.797
2- to 21-week	ENSRNOG00000020269	SURP and G patch domain containing 2(Sugp2)	0.824
2- to 21-week	ENSRNOG00000020344	syntrophin, beta 2(Sntb2)	0.791
2- to 21-week	ENSRNOG00000020347	nucleoporin 98(Nup98)	0.685
2- to 21-week	ENSRNOG00000020500	CDC-like kinase 2(Clk2)	0.744
2- to 21-week	ENSRNOG00000020689	cytoplasmic polyadenylation element binding protein 3(Cpeb3)	0.767
2- to 21-week	ENSRNOG00000020748	microtubule-associated protein 4(Map4)	0.844
2- to 21-week	ENSRNOG00000020769	CREB3 regulatory factor(Crebrf)	0.798
2- to 21-week	ENSRNOG00000021203	atlastin GTPase 3(Atl3)	0.651
2- to 21-week	ENSRNOG00000021569	T-cell lymphoma invasion and metastasis 1(Tiam1)	0.692
2- to 21-week	ENSRNOG00000022066	family with sequence similarity 117, member B(Fam117b)	0.686
2- to 21-week	ENSRNOG00000022343	ALMS1, centrosome and basal body associated protein(Alms1)	0.739
2- to 21-week	ENSRNOG00000022445	IQ motif containing F3(Iqcf3)	0.699
2- to 21-week	ENSRNOG00000022845	centrosomal protein 70(Cep70)	0.699
2- to 21-week	ENSRNOG00000023202	ubiquitin specific peptidase 15(Usp15)	0.933
2- to 21-week	ENSRNOG00000023566	von Willebrand factor A domain containing 3B(Vwa3b)	0.692
2- to 21-week	ENSRNOG00000023807	cyclin Y-like 1(Ccnyl1)	0.736
2- to 21-week	ENSRNOG00000023941	family with sequence similarity 178, member B(Fam178b)	0.725
2- to 21-week	ENSRNOG00000024272	INO80 complex subunit D(Ino80d)	0.699
2- to 21-week	ENSRNOG00000024402	focadhesin(Focad)	0.737
2- to 21-week	ENSRNOG00000024521	spermatogenesis associated 16(Spata16)	0.699

Age	Ensembl ID	Gene name	*ρ*
2- to 21-week	ENSRNOG00000024530	LYR motif containing 1(Lyrm1)	0.679
2- to 21-week	ENSRNOG00000024563	glucosidase, alpha; neutral C(Ganc)	0.857
2- to 21-week	ENSRNOG00000024632	activating transcription factor 6(Atf6)	0.758
2- to 21-week	ENSRNOG00000024808	serine threonine kinase 39(Stk39)	0.650
2- to 21-week	ENSRNOG00000024837	transmembrane phosphoinositide 3-phosphatase and tensin homolog 2(Tpte2)	0.824
2- to 21-week	ENSRNOG00000024870	ankyrin repeat and sterile alpha motif domain containing 1B(Anks1b)	0.651
2- to 21-week	ENSRNOG00000024878	RNA binding motif protein 44(Rbm44)	0.797
2- to 21-week	ENSRNOG00000025141	kizuna centrosomal protein(Kiz)	0.679
2- to 21-week	ENSRNOG00000025332	CD109 molecule(Cd109)	0.699
2- to 21-week	ENSRNOG00000025406	IQ motif containing GTPase activating protein 2(Iqgap2)	0.699
2- to 21-week	ENSRNOG00000025551	regulator of G-protein signaling 22(Rgs22)	0.789
2- to 21-week	ENSRNOG00000025728	ADAM metallopeptidase domain 32(Adam32)	0.821
2- to 21-week	ENSRNOG00000025787	sperm associated antigen 6(Spag6)	0.844
2- to 21-week	ENSRNOG00000026589	dpy-19 like 1(Dpy19l1)	0.725
2- to 21-week	ENSRNOG00000026610	akirin 1(Akirin1)	0.699
2- to 21-week	ENSRNOG00000026842	nicotinamide nucleotide transhydrogenase(Nnt)	0.616
2- to 21-week	ENSRNOG00000026845	family with sequence similarity 81, member B(Fam81b)	0.699
2- to 21-week	ENSRNOG00000027012	ubiquitin specific peptidase 54(Usp54)	0.857
2- to 21-week	ENSRNOG00000027617	tetratricopeptide repeat domain 26(Ttc26)	0.705
2- to 21-week	ENSRNOG00000027801	zona pellucida binding protein(Zpbp)	0.711
2- to 21-week	ENSRNOG00000027992	DS cell adhesion molecule(Dscam)	0.699
2- to 21-week	ENSRNOG00000028501	zinc finger CCCH-type containing 18(Zc3h18)	0.699
2- to 21-week	ENSRNOG00000029773	ATM serine/threonine kinase(Atm)	0.674
2- to 21-week	ENSRNOG00000031038	glycosyltransferase-like domain containing 1(Gtdc1)	0.757
2- to 21-week	ENSRNOG00000031247	leucine, glutamate and lysine rich 1(Lekr1)	0.884
2- to 21-week	ENSRNOG00000032570	structural maintenance of chromosomes 1B(Smc1b)	0.699
2- to 21-week	ENSRNOG00000033169	cytoplasmic polyadenylation element binding protein 4(Cpeb4)	0.699
2- to 21-week	ENSRNOG00000033528	tolloid-like 1(Tll1)	0.889
2- to 21-week	ENSRNOG00000036576	zinc finger, DHHC-type containing 6(Zdhhc6)	0.705
2- to 21-week	ENSRNOG00000036675	BTB domain containing 16(Btbd16)	0.686
2- to 21-week	ENSRNOG00000036964	Ral GTPase activating protein catalytic alpha subunit 2(Ralgapa2)	0.774
2- to 21-week	ENSRNOG00000037865	HORMA domain containing 2(Hormad2)	0.631
2- to 21-week	ENSRNOG00000038242	similar to T-cell activation Rho GTPase-activating protein isoform b(LOC365791)	0.783
2- to 21-week	ENSRNOG00000039300	adenosylhomocysteinase-like 2(Ahcyl2)	0.679
2- to 21-week	ENSRNOG00000042068	OTU deubiquitinase 7B(Otud7b)	0.725
2- to 21-week	ENSRNOG00000042432	NIMA-related kinase 11(Nek11)	0.674
2- to 21-week	ENSRNOG00000042879	SEH1-like (S. cerevisiae)(Seh1l)	0.613
2- to 21-week	ENSRNOG00000043035	F-box and leucine-rich repeat protein 13(Fbxl13)	0.626
2- to 21-week	ENSRNOG00000045585	dpy-19 like 2(Dpy19l2)	0.722
2- to 21-week	ENSRNOG00000046023	exocyst complex component 4(Exoc4)	0.677
2- to 21-week	ENSRNOG00000048719	dystrobrevin binding protein 1(Dtnbp1)	0.828

Age	Ensembl ID	Gene name	*ρ*
2- to 21-week	ENSRNOG00000049507	septin 10(Sept10)	0.647
2- to 21-week	ENSRNOG00000050191	coiled-coil domain containing 7(Ccdc7)	0.692
2- to 21-week	ENSRNOG00000052688	dynein, axonemal, heavy chain 2(Dnah2)	0.616
2- to 21-week	ENSRNOG00000053060	zinc finger, C3H1-type containing(Zfc3h1)	0.616
2- to 21-week	ENSRNOG00000054331	WD repeat containing planar cell polarity effector(Wdpcp)	0.681
2- to 21-week	ENSRNOG00000054901	replication timing regulatory factor 1(Rif1)	0.699
2- to 21-week	ENSRNOG00000056330	cyclic nucleotide binding domain containing 1(Cnbd1)	0.615
2- to 21-week	ENSRNOG00000056647	activating signal cointegrator 1 complex subunit 1(Ascc1)	0.651
2- to 21-week	ENSRNOG00000056826	ArfGAP with RhoGAP domain, ankyrin repeat and PH domain 2(Arap2)	0.651
2- to 21-week	ENSRNOG00000057042	propionyl-CoA carboxylase alpha subunit(Pcca)	0.621
2- to 21-week	ENSRNOG00000057315	potassium voltage-gated channel subfamily H member 3(Kcnh3)	0.632
2- to 21-week	ENSRNOG00000057372	methyltransferase like 6(Mettl6)	0.857
2- to 21-week	ENSRNOG00000057740	coiled-coil domain containing 148(Ccdc148)	0.843
2- to 21-week	ENSRNOG00000057815	coiled-coil domain containing 191(Ccdc191)	0.774
2- to 21-week	ENSRNOG00000057945	Fanconi anemia, complementation group G(Fancg)	0.710
2- to 21-week	ENSRNOG00000058275	sperm flagellar 2(Spef2)	0.659
2- to 21-week	ENSRNOG00000058805	axin interactor, dorsalization associated(Aida)	0.659
2- to 21-week	ENSRNOG00000059276	similar to hypothetical protein FLJ10706(LOC498265)	0.844
2- to 21-week	ENSRNOG00000059659	zinc finger AN1-type containing 3(Zfand3)	0.699
2- to 21-week	ENSRNOG00000059865	dynein, axonemal, heavy chain 12(Dnah12)	0.828
2- to 21-week	ENSRNOG00000060561	glycerophosphodiester phosphodiesterase domain containing 1(Gdpd1)	0.828
2- to 21-week	ENSRNOG00000060984	dynein, axonemal, heavy chain 7(Dnah7)	0.813
2- to 21-week	ENSRNOG00000061040	HECT and RLD domain containing E3 ubiquitin protein ligase 4(Herc4)	0.617
2- to 21-week	ENSRNOG00000061424	mitogen activated protein kinase kinase kinase 3(Map3k3)	0.616
2- to 21-week	ENSRNOG00000061543	adaptor-related protein complex 2, beta 1 subunit(Ap2b1)	0.699
2- to 21-week	ENSRNOG00000061795	RNA binding motif protein 4B(Rbm4b)	0.644
21- to 104-week	ENSRNOG00000000042	xenotropic and polytropic retrovirus receptor 1(Xpr1)	-0.750
21- to 104-week	ENSRNOG00000000150	DnaJ heat shock protein family (Hsp40) member C1(Dnajc1)	-0.659
21- to 104-week	ENSRNOG00000000262	zinc finger protein 821(Zfp821)	-0.930
21- to 104-week	ENSRNOG00000000303	centrosomal protein 57-like 1(Cep57l1)	-0.750
21- to 104-week	ENSRNOG00000000540	BTB domain containing 9(Btbd9)	-0.930
21- to 104-week	ENSRNOG00000000542	dynein, axonemal, heavy chain 8(Dnah8)	-0.930
21- to 104-week	ENSRNOG00000000593	REV3 like, DNA directed polymerase zeta catalytic subunit(Rev3l)	-0.639
21- to 104-week	ENSRNOG00000000802	TBC1 domain family, member 32(Tbc1d32)	-0.930
21- to 104-week	ENSRNOG00000001178	signal peptide peptidase like 3(Sppl3)	-0.750
21- to 104-week	ENSRNOG00000001294	intraflagellar transport 81(Ift81)	-0.750
21- to 104-week	ENSRNOG00000001971	BBX, HMG-box containing(Bbx)	-0.873
21- to 104-week	ENSRNOG00000002112	zinc finger protein 644(Zfp644)	-0.671
21- to 104-week	ENSRNOG00000002142	CDP-diacylglycerol synthase 1(Cds1)	-0.930
21- to 104-week	ENSRNOG00000002176	nectin cell adhesion molecule 3(Nectin3)	-0.639
21- to 104-week	ENSRNOG00000002204	coiled-coil domain containing 158(Ccdc158)	-0.750

Age	Ensembl ID	Gene name	*ρ*
21- to 104-week	ENSRNOG00000002275	factor interacting with PAPOLA and CPSF1(Fip1l1)	-0.750
21- to 104-week	ENSRNOG00000002496	syntaxin binding protein 5-like(Stxbp5l)	-0.930
21- to 104-week	ENSRNOG00000002504	spermatogenesis associated 17(Spata17)	-0.750
21- to 104-week	ENSRNOG00000002516	myotubularin 1(Mtm1)	-0.894
21- to 104-week	ENSRNOG00000002532	protamine 1(Prm1)	-0.750
21- to 104-week	ENSRNOG00000002538	erythrocyte membrane protein band 4.1 like 5(Epb41l5)	-0.930
21- to 104-week	ENSRNOG00000002544	kinesin-associated protein 3(Kifap3)	-0.930
21- to 104-week	ENSRNOG00000002697	myotubularin related protein 1(Mtmr1)	-0.930
21- to 104-week	ENSRNOG00000002736	RAB GTPase activating protein 1-like(Rabgap1l)	-0.930
21- to 104-week	ENSRNOG00000002755	platelet-activating factor acetylhydrolase 1b, regulatory subunit 1(Pafah1b1)	-0.873
21- to 104-week	ENSRNOG00000002778	apoptosis antagonizing transcription factor(Aatf)	-0.639
21- to 104-week	ENSRNOG00000002855	replication factor C subunit 1(Rfc1)	-0.750
21- to 104-week	ENSRNOG00000002882	zinc finger CCHC-type containing 2(Zcchc2)	-0.878
21- to 104-week	ENSRNOG00000003078	DDB1 and CUL4 associated factor 6(Dcaf6)	-0.894
21- to 104-week	ENSRNOG00000003186	ankyrin repeat and sterile alpha motif domain containing 3(Anks3)	-0.930
21- to 104-week	ENSRNOG00000003213	helicase with zinc finger(Helz)	-0.750
21- to 104-week	ENSRNOG00000003258	cell division cycle 73(Cdc73)	-0.930
21- to 104-week	ENSRNOG00000003273	spermatogenesis associated 20(Spata20)	-0.930
21- to 104-week	ENSRNOG00000003550	acyl-CoA binding domain containing 6(Acbd6)	-0.873
21- to 104-week	ENSRNOG00000003695	MGAT4 family, member D(Mgat4d)	-0.894
21- to 104-week	ENSRNOG00000003908	centrosomal protein 128(Cep128)	-0.873
21- to 104-week	ENSRNOG00000003955	spermatogenesis associated 7(Spata7)	-0.750
21- to 104-week	ENSRNOG00000003979	zinc finger RANBP2-type containing 3(Zranb3)	-0.639
21- to 104-week	ENSRNOG00000003991	ankyrin and armadillo repeat containing(Ankar)	-0.930
21- to 104-week	ENSRNOG00000004076	PMS1 homolog 1, mismatch repair system component(Pms1)	-0.750
21- to 104-week	ENSRNOG00000004085	T cell activation inhibitor, mitochondrial(Tcaim)	-0.750
21- to 104-week	ENSRNOG00000004181	serologically defined colon cancer antigen 8(Sdccag8)	-0.750
21- to 104-week	ENSRNOG00000004185	MON2 homolog, regulator of endosome-to-Golgi trafficking(Mon2)	-0.750
21- to 104-week	ENSRNOG00000004201	regulating synaptic membrane exocytosis 2(Rims2)	-0.873
21- to 104-week	ENSRNOG00000004290	growth factor receptor bound protein 10(Grb10)	-0.930
21- to 104-week	ENSRNOG00000004364	brain and reproductive organ-expressed (TNFRSF1A modulator)(Bre)	-0.783
21- to 104-week	ENSRNOG00000004456	clathrin heavy chain linker domain containing 1(Clhc1)	-0.750
21- to 104-week	ENSRNOG00000004493	taspase 1(Tasp1)	-0.639
21- to 104-week	ENSRNOG00000004534	speedy/RINGO cell cycle regulator family member A(Spdya)	-0.750
21- to 104-week	ENSRNOG00000004564	leucine-rich repeats and IQ motif containing 1(Lrriq1)	-0.930
21- to 104-week	ENSRNOG00000004585	transmembrane and tetratricopeptide repeat containing 2(Tmtc2)	-0.639
21- to 104-week	ENSRNOG00000004621	reticulon 4(Rtn4)	-0.930
21- to 104-week	ENSRNOG00000004682	PARP1 binding protein(Parpbp)	-0.930
21- to 104-week	ENSRNOG00000004736	Ral GEF with PH domain and SH3 binding motif 2(Ralgps2)	-0.873
21- to 104-week	ENSRNOG00000004826	SOS Ras/Rho guanine nucleotide exchange factor 2(Sos2)	-0.671
21- to 104-week	ENSRNOG00000004908	structural maintenance of chromosomes 6(Smc6)	-0.639

Age	Ensembl ID	Gene name	*ρ*
21- to 104-week	ENSRNOG00000005042	RAN binding protein 17(Ranbp17)	-0.894
21- to 104-week	ENSRNOG00000005081	F-box and leucine-rich repeat protein 20(Fbxl20)	-0.750
21- to 104-week	ENSRNOG00000005101	cutaneous T-cell lymphoma-associated antigen 5 homolog(LOC100912115)	-0.750
21- to 104-week	ENSRNOG00000005105	Sad1 and UNC84 domain containing 3(Sun3)	-0.639
21- to 104-week	ENSRNOG00000005233	leucine rich repeat containing 6(Lrrc6)	-0.930
21- to 104-week	ENSRNOG00000005433	SHQ1, H/ACA ribonucleoprotein assembly factor(Shq1)	-0.639
21- to 104-week	ENSRNOG00000005907	RAD18 E3 ubiquitin protein ligase(Rad18)	-0.930
21- to 104-week	ENSRNOG00000005916	protein phosphatase, Mg2+/Mn2+ dependent, 1A(Ppm1a)	-0.750
21- to 104-week	ENSRNOG00000005927	cleavage and polyadenylation specific factor 6(Cpsf6)	-0.894
21- to 104-week	ENSRNOG00000005934	muscular LMNA-interacting protein(Mlip)	-0.930
21- to 104-week	ENSRNOG00000006001	LUC7-like 2 pre-mRNA splicing factor(Luc7l2)	-0.930
21- to 104-week	ENSRNOG00000006002	serine/threonine kinase 35(Stk35)	-0.750
21- to 104-week	ENSRNOG00000006114	cilia and flagella associated protein 69(Cfap69)	-0.750
21- to 104-week	ENSRNOG00000006136	3-phosphoinositide dependent protein kinase-1(Pdpk1)	-0.750
21- to 104-week	ENSRNOG00000006180	pumilio RNA-binding family member 2(Pum2)	-0.750
21- to 104-week	ENSRNOG00000006235	neural EGFL like 2(Nell2)	-0.750
21- to 104-week	ENSRNOG00000006248	tripartite motif-containing 37(Trim37)	-0.873
21- to 104-week	ENSRNOG00000006304	MDM2 proto-oncogene(Mdm2)	-0.894
21- to 104-week	ENSRNOG00000006340	mediator of cell motility 1(Memo1)	-0.878
21- to 104-week	ENSRNOG00000006785	DDB1 and CUL4 associated factor 8(Dcaf8)	-0.783
21- to 104-week	ENSRNOG00000006816	ubiquitin protein ligase E3 component n-recognin 5(Ubr5)	-0.873
21- to 104-week	ENSRNOG00000006828	bromodomain adjacent to zinc finger domain, 1A(Baz1a)	-0.750
21- to 104-week	ENSRNOG00000006832	zinc finger, DHHC-type containing 5(Zdhhc5)	-0.873
21- to 104-week	ENSRNOG00000007054	DEAH-box helicase 33(Dhx33)	-0.750
21- to 104-week	ENSRNOG00000007125	VPS54 GARP complex subunit(Vps54)	-0.930
21- to 104-week	ENSRNOG00000007127	Bardet-Biedl syndrome 5(Bbs5)	-0.894
21- to 104-week	ENSRNOG00000007341	maestro heat-like repeat family member 8(Mroh8)	-0.750
21- to 104-week	ENSRNOG00000007430	SLX4 interacting protein(Slx4ip)	-0.750
21- to 104-week	ENSRNOG00000007523	chaperonin containing TCP1 subunit 6B(Cct6b)	-0.930
21- to 104-week	ENSRNOG00000007568	spermatogenesis associated 6(Spata6)	-0.639
21- to 104-week	ENSRNOG00000007646	signal-induced proliferation-associated 1 like 1(Sipa1l1)	-0.639
21- to 104-week	ENSRNOG00000007730	vomeronasal 2 receptor, 53(Vom2r53)	-0.930
21- to 104-week	ENSRNOG00000007748	testis expressed 15(Tex15)	-0.930
21- to 104-week	ENSRNOG00000007867	RNA guanylyltransferase and 5'-phosphatase(Rngtt)	-0.930
21- to 104-week	ENSRNOG00000008034	transmembrane protein with EGF-like and two follistatin-like domains 1(Tmeff1)	-0.878
21- to 104-week	ENSRNOG00000008053	ATPase phospholipid transporting 8A2(Atp8a2)	-0.930
21- to 104-week	ENSRNOG00000008075	intraflagellar transport 74(Ift74)	-0.639
21- to 104-week	ENSRNOG00000008393	Tax1 binding protein 1(Tax1bp1)	-0.930
21- to 104-week	ENSRNOG00000008410	centrosomal protein 63(Cep63)	-0.930
21- to 104-week	ENSRNOG00000008463	RUN domain containing 3B(Rundc3b)	-0.639
21- to 104-week	ENSRNOG00000008524	glucocorticoid induced 1(Glcci1)	-0.750

Age	Ensembl ID	Gene name	ρ
21- to 104-week	ENSRNOG00000008775	leucine-rich repeats and guanylate kinase domain containing(Lrguk)	-0.930
21- to 104-week	ENSRNOG00000008806	CAP-GLY domain containing linker protein family, member 4(Clip4)	-0.750
21- to 104-week	ENSRNOG00000008859	TRAF family member-associated NFKB activator(Tank)	-0.878
21- to 104-week	ENSRNOG00000008869	protein phosphatase 1, regulatory subunit 9A(Ppp1r9a)	-0.750
21- to 104-week	ENSRNOG00000008929	AE binding protein 2(Aebp2)	-0.930
21- to 104-week	ENSRNOG00000008986	diaphanous-related formin 3(Diaph3)	-0.878
21- to 104-week	ENSRNOG00000009034	tudor domain containing 3(Tdrd3)	-0.750
21- to 104-week	ENSRNOG00000009208	progesterone immunomodulatory binding factor 1(Pibf1)	-0.930
21- to 104-week	ENSRNOG00000009264	ELKS/RAB6-interacting/CAST family member 1(Erc1)	-0.873
21- to 104-week	ENSRNOG00000009278	intraflagellar transport 88(Ift88)	-0.930
21- to 104-week	ENSRNOG00000009303	zwilch kinetochore protein(Zwilch)	-0.768
21- to 104-week	ENSRNOG00000009318	tubulin tyrosine ligase like 5(Ttll5)	-0.878
21- to 104-week	ENSRNOG00000009338	KRAS proto-oncogene, GTPase(Kras)	-0.930
21- to 104-week	ENSRNOG00000009539	KH RNA binding domain containing, signal transduction associated 3(Khdrbs3)	-0.639
21- to 104-week	ENSRNOG00000009555	leucine-rich repeats and IQ motif containing 3(Lrriq3)	-0.930
21- to 104-week	ENSRNOG00000009585	transcription factor 20(Tcf20)	-0.873
21- to 104-week	ENSRNOG00000009664	ankyrin repeat domain 42(Ankrd42)	-0.894
21- to 104-week	ENSRNOG00000009690	ring finger protein 219(Rnf219)	-0.750
21- to 104-week	ENSRNOG00000009745	protein phosphatase 3 catalytic subunit gamma(Ppp3cc)	-0.873
21- to 104-week	ENSRNOG00000009766	G protein-coupled receptor 180(Gpr180)	-0.750
21- to 104-week	ENSRNOG00000009792	zinc finger, B-box domain containing(Zbbx)	-0.930
21- to 104-week	ENSRNOG00000009808	intraflagellar transport 80(Ift80)	-0.894
21- to 104-week	ENSRNOG00000009812	SET binding factor 2(Sbf2)	-0.894
21- to 104-week	ENSRNOG00000009851	solute carrier family 38, member 9(Slc38a9)	-0.873
21- to 104-week	ENSRNOG00000009930	phosphatidylinositol glycan anchor biosynthesis, class O(Pigo)	-0.639
21- to 104-week	ENSRNOG00000009956	WNK lysine deficient protein kinase 1(Wnk1)	-0.659
21- to 104-week	ENSRNOG00000010024	cytochrome b5 reductase 4(Cyb5r4)	-0.878
21- to 104-week	ENSRNOG00000010027	ATR serine/threonine kinase(Atr)	-0.894
21- to 104-week	ENSRNOG00000010150	spermatid perinuclear RNA binding protein(Strbp)	-0.873
21- to 104-week	ENSRNOG00000010155	pericentriolar material 1(Pcm1)	-0.659
21- to 104-week	ENSRNOG00000010418	NIMA-related kinase 1(Nek1)	-0.873
21- to 104-week	ENSRNOG00000010479	MYC binding protein 2, E3 ubiquitin protein ligase(Mycbp2)	-0.878
21- to 104-week	ENSRNOG00000010517	family with sequence similarity 126, member A(Fam126a)	-0.878
21- to 104-week	ENSRNOG00000010526	DENN domain containing 1A(Dennd1a)	-0.878
21- to 104-week	ENSRNOG00000010536	nuclear receptor subfamily 2, group C, member 2(Nr2c2)	-0.750
21- to 104-week	ENSRNOG00000010567	angel homolog 1(Angel1)	-0.930
21- to 104-week	ENSRNOG00000010624	adaptor-related protein complex 3, beta 1 subunit(Ap3b1)	-0.768
21- to 104-week	ENSRNOG00000010637	TBC1 domain family, member 5(Tbc1d5)	-0.873
21- to 104-week	ENSRNOG00000010815	E74-like factor 2(Elf2)	-0.894
21- to 104-week	ENSRNOG00000010819	heat shock protein 4-like(Hspa4l)	-0.894
21- to 104-week	ENSRNOG00000010952	intraflagellar transport 122(Ift122)	-0.750

Age	Ensembl ID	Gene name	*ρ*
21- to 104-week	ENSRNOG00000010957	B-Raf proto-oncogene, serine/threonine kinase(Braf)	-0.750
21- to 104-week	ENSRNOG00000011000	regulating synaptic membrane exocytosis 1(Rims1)	-0.894
21- to 104-week	ENSRNOG00000011024	zinc finger, DHHC-type containing 20(Zdhhc20)	-0.750
21- to 104-week	ENSRNOG00000011059	tau tubulin kinase 2(Ttbk2)	-0.894
21- to 104-week	ENSRNOG00000011063	DENN domain containing 1B(Dennd1b)	-0.873
21- to 104-week	ENSRNOG00000011066	membrane associated ring-CH-type finger 6(March6)	-0.930
21- to 104-week	ENSRNOG00000011094	EF-hand calcium binding domain 6(Efcab6)	-0.750
21- to 104-week	ENSRNOG00000011199	poly (ADP-ribose) polymerase family, member 6(Parp6)	-0.639
21- to 104-week	ENSRNOG00000011307	sarcolemma associated protein(Slmap)	-0.764
21- to 104-week	ENSRNOG00000011376	basal body orientation factor 1(Bbof1)	-0.750
21- to 104-week	ENSRNOG00000011417	phosphodiesterase 3B(Pde3b)	-0.894
21- to 104-week	ENSRNOG00000011453	kinesin family member 6(Kif6)	-0.750
21- to 104-week	ENSRNOG00000011603	calcium binding protein 39-like(Cab39l)	-0.750
21- to 104-week	ENSRNOG00000011619	myosin IXA(Myo9a)	-0.873
21- to 104-week	ENSRNOG00000011625	tankyrase(Tnks)	-0.750
21- to 104-week	ENSRNOG00000011627	zinc finger RNA binding protein(Zfr)	-0.783
21- to 104-week	ENSRNOG00000011669	glutathione S-transferase, C-terminal domain containing(Gstcd)	-0.930
21- to 104-week	ENSRNOG00000011791	zinc finger AN1-type containing 4(Zfand4)	-0.930
21- to 104-week	ENSRNOG00000011847	G protein-coupled receptor kinase 4(Grk4)	-0.878
21- to 104-week	ENSRNOG00000012015	leucine rich repeat containing 49(Lrrc49)	-0.639
21- to 104-week	ENSRNOG00000012055	mannosidase, alpha, class 2A, member 2(Man2a2)	-0.894
21- to 104-week	ENSRNOG00000012237	radixin(Rdx)	-0.750
21- to 104-week	ENSRNOG00000012325	adenosine kinase(Adk)	-0.930
21- to 104-week	ENSRNOG00000012486	primase (DNA) subunit 2(Prim2)	-0.750
21- to 104-week	ENSRNOG00000012536	sphingomyelin synthase 1(Sgms1)	-0.878
21- to 104-week	ENSRNOG00000012637	amyotrophic lateral sclerosis 2 chromosome region, candidate 11(Als2cr11)	-0.750
21- to 104-week	ENSRNOG00000012655	ADAM metallopeptidase with thrombospondin type 1 motif, 6(Adamts6)	-0.930
21- to 104-week	ENSRNOG00000012719	tudor domain containing 12(Tdrd12)	-0.930
21- to 104-week	ENSRNOG00000013061	tumor suppressor candidate 3(Tusc3)	-0.639
21- to 104-week	ENSRNOG00000013293	methylcrotonoyl-CoA carboxylase 1(Mccc1)	-0.750
21- to 104-week	ENSRNOG00000013318	coiled-coil domain containing 150(Ccdc150)	-0.894
21- to 104-week	ENSRNOG00000013322	DNA polymerase alpha 1, catalytic subunit(Pola1)	-0.639
21- to 104-week	ENSRNOG00000013529	serine/threonine kinase 4(Stk4)	-0.894
21- to 104-week	ENSRNOG00000013533	apoptotic chromatin condensation inducer 1(Acin1)	-0.750
21- to 104-week	ENSRNOG00000013780	neurofibromin 1(Nf1)	-0.878
21- to 104-week	ENSRNOG00000013962	membrane associated guanylate kinase, WW and PDZ domain containing 2(Magi2)	-0.750
21- to 104-week	ENSRNOG00000014139	sodium channel and clathrin linker 1(Sclt1)	-0.878
21- to 104-week	ENSRNOG00000014163	zinc finger protein 536(Zfp536)	-0.750
21- to 104-week	ENSRNOG00000014248	erb-b2 receptor tyrosine kinase 4(Erbb4)	-0.750
21- to 104-week	ENSRNOG00000014276	phospholipase C, epsilon 1(Plce1)	-0.750
21- to 104-week	ENSRNOG00000014368	eukaryotic translation initiation factor 4 gamma, 3(Eif4g3)	-0.878

Age	Ensembl ID	Gene name	ρ
21- to 104-week	ENSRNOG00000014486	regulatory factor X3(Rfx3)	-0.894
21- to 104-week	ENSRNOG00000014513	exonuclease NEF-sp(Exnef)	-0.894
21- to 104-week	ENSRNOG00000014576	dedicator of cyto-kinesis 3(Dock3)	-0.873
21- to 104-week	ENSRNOG00000014584	outer dense fiber of sperm tails 2(Odf2)	-0.750
21- to 104-week	ENSRNOG00000014590	serine/threonine kinase 33(Stk33)	-0.750
21- to 104-week	ENSRNOG00000014670	zinc finger, MYND-type containing 11(Zmynd11)	-0.750
21- to 104-week	ENSRNOG00000014775	nucleolar protein 4(Nol4)	-0.930
21- to 104-week	ENSRNOG00000014875	ASH2 like histone lysine methyltransferase complex subunit(Ash2l)	-0.894
21- to 104-week	ENSRNOG00000014893	WD repeat domain 63(Wdr63)	-0.750
21- to 104-week	ENSRNOG00000015097	potassium channel modulatory factor 1(Kcmf1)	-0.750
21- to 104-week	ENSRNOG00000015189	Bardet-Biedl syndrome 9(Bbs9)	-0.873
21- to 104-week	ENSRNOG00000015288	disco-interacting protein 2 homolog C(Dip2c)	-0.750
21- to 104-week	ENSRNOG00000015339	SHOC2 leucine-rich repeat scaffold protein(Shoc2)	-0.750
21- to 104-week	ENSRNOG00000015357	BMP-binding endothelial regulator(Bmper)	-0.930
21- to 104-week	ENSRNOG00000015440	Werner syndrome RecQ like helicase(Wrn)	-0.750
21- to 104-week	ENSRNOG00000015528	praja ring finger ubiquitin ligase 2(Pja2)	-0.750
21- to 104-week	ENSRNOG00000015734	ubiquitin protein ligase E3A(Ube3a)	-0.894
21- to 104-week	ENSRNOG00000015783	ATP-binding cassette, subfamily A (ABC1), member 16(Abca16)	-0.750
21- to 104-week	ENSRNOG00000015898	FER tyrosine kinase(Fer)	-0.878
21- to 104-week	ENSRNOG00000015977	zinc finger protein 609(Zfp609)	-0.659
21- to 104-week	ENSRNOG00000016121	thyroid hormone receptor interactor 4(Trip4)	-0.750
21- to 104-week	ENSRNOG00000016133	small ubiquitin-like modifier 1(Sumo1)	-0.750
21- to 104-week	ENSRNOG00000016152	DEK proto-oncogene(Dek)	-0.750
21- to 104-week	ENSRNOG00000016183	intracisternal A particle-promoted polypeptide(Ipp)	-0.671
21- to 104-week	ENSRNOG00000016360	G protein pathway suppressor 2(Gps2)	-0.930
21- to 104-week	ENSRNOG00000016454	nuclear autoantigenic sperm protein(Nasp)	-0.750
21- to 104-week	ENSRNOG00000016545	intraflagellar transport 140(Ift140)	-0.750
21- to 104-week	ENSRNOG00000016620	casein kinase 1, gamma 1(Csnk1g1)	-0.894
21- to 104-week	ENSRNOG00000016635	similar to K04F10.2(LOC361646)	-0.750
21- to 104-week	ENSRNOG00000016703	general transcription factor 2A subunit 1 like(Gtf2a1l)	-0.930
21- to 104-week	ENSRNOG00000016717	growth arrest-specific 2(Gas2)	-0.894
21- to 104-week	ENSRNOG00000016722	exportin 6(Xpo6)	-0.750
21- to 104-week	ENSRNOG00000016815	transmembrane protein 135(Tmem135)	-0.750
21- to 104-week	ENSRNOG00000016835	synaptonemal complex protein 1(Sycp1)	-0.873
21- to 104-week	ENSRNOG00000016910	Ral GEF with PH domain and SH3 binding motif 1(Ralgps1)	-0.894
21- to 104-week	ENSRNOG00000016963	thyroid hormone receptor interactor 12(Trip12)	-0.671
21- to 104-week	ENSRNOG00000016992	POTE ankyrin domain family, member C(Potec)	-0.930
21- to 104-week	ENSRNOG00000017074	RAB28, member RAS oncogene family(Rab28)	-0.750
21- to 104-week	ENSRNOG00000017178	Hydin, axonemal central pair apparatus protein(Hydin)	-0.930
21- to 104-week	ENSRNOG00000017282	testis-specific kinase 2(Tesk2)	-0.930
21- to 104-week	ENSRNOG00000017505	aminopeptidase O(Npepo)	-0.659

Age	Ensembl ID	Gene name	*ρ*
21- to 104-week	ENSRNOG00000017518	ADAM metallopeptidase domain 5(Adam5)	-0.930
21- to 104-week	ENSRNOG00000017554	ADAM metallopeptidase domain 3A(Adam3a)	-0.930
21- to 104-week	ENSRNOG00000017581	similar to 4833420G17Rik protein(RGD1306227)	-0.750
21- to 104-week	ENSRNOG00000017608	C2 calcium-dependent domain containing 3(C2cd3)	-0.930
21- to 104-week	ENSRNOG00000017781	DnaJ heat shock protein family (Hsp40) member C7(Dnajc7)	-0.750
21- to 104-week	ENSRNOG00000017824	nuclear receptor coactivator 5(Ncoa5)	-0.878
21- to 104-week	ENSRNOG00000017883	calmodulin regulated spectrin-associated protein 1(Camsap1)	-0.873
21- to 104-week	ENSRNOG00000018382	inositol polyphosphate-4-phosphatase type II B(Inpp4b)	-0.930
21- to 104-week	ENSRNOG00000018387	WD repeat domain 7(Wdr7)	-0.764
21- to 104-week	ENSRNOG00000018404	alanyl-tRNA synthetase(Aars)	-0.639
21- to 104-week	ENSRNOG00000018425	dymeclin(Dym)	-0.783
21- to 104-week	ENSRNOG00000018457	protein phosphatase 2 phosphatase activator(Ptpa)	-0.750
21- to 104-week	ENSRNOG00000018507	glutamine fructose-6-phosphate transaminase 1(Gfpt1)	-0.639
21- to 104-week	ENSRNOG00000018529	casein kinase 1, gamma 2(Csnk1g2)	-0.930
21- to 104-week	ENSRNOG00000018651	ATP/GTP binding protein 1(Agtpbp1)	-0.655
21- to 104-week	ENSRNOG00000018946	tripartite motif-containing 33(Trim33)	-0.878
21- to 104-week	ENSRNOG00000018996	PHD finger protein 7(Phf7)	-0.750
21- to 104-week	ENSRNOG00000019020	Bardet-Biedl syndrome 2(Bbs2)	-0.930
21- to 104-week	ENSRNOG00000019069	nuclear RNA export factor 1(Nxf1)	-0.783
21- to 104-week	ENSRNOG00000019105	dipeptidylpeptidase 8(Dpp8)	-0.783
21- to 104-week	ENSRNOG00000019271	unc-51 like kinase 4(Ulk4)	-0.930
21- to 104-week	ENSRNOG00000019272	G kinase anchoring protein 1(Gkap1)	-0.894
21- to 104-week	ENSRNOG00000019671	round spermatid basic protein 1(Rsbn1)	-0.639
21- to 104-week	ENSRNOG00000019752	solute carrier family 29 member 1(Slc29a1)	-0.930
21- to 104-week	ENSRNOG00000019826	SIL1 nucleotide exchange factor(Sil1)	-0.768
21- to 104-week	ENSRNOG00000020118	NME/NM23 family member 5(Nme5)	-0.930
21- to 104-week	ENSRNOG00000020191	TBC1 domain family, member 17(Tbc1d17)	-0.750
21- to 104-week	ENSRNOG00000020203	protein tyrosine phosphatase, non-receptor type 20(Ptpn20)	-0.930
21- to 104-week	ENSRNOG00000020269	SURP and G patch domain containing 2(Sugp2)	-0.930
21- to 104-week	ENSRNOG00000020344	syntrophin, beta 2(Sntb2)	-0.894
21- to 104-week	ENSRNOG00000020425	stromal interaction molecule 1(Stim1)	-0.750
21- to 104-week	ENSRNOG00000020500	CDC-like kinase 2(Clk2)	-0.894
21- to 104-week	ENSRNOG00000020514	SRY box 6(Sox6)	-0.930
21- to 104-week	ENSRNOG00000020689	cytoplasmic polyadenylation element binding protein 3(Cpeb3)	-0.878
21- to 104-week	ENSRNOG00000020748	microtubule-associated protein 4(Map4)	-0.930
21- to 104-week	ENSRNOG00000020915	SET domain containing 2(Setd2)	-0.894
21- to 104-week	ENSRNOG00000021044	G patch domain containing 8(Gpatch8)	-0.750
21- to 104-week	ENSRNOG00000021203	atlastin GTPase 3(Atl3)	-0.639
21- to 104-week	ENSRNOG00000021222	VPS16 CORVET/HOPS core subunit(Vps16)	-0.750
21- to 104-week	ENSRNOG00000021497	AKT serine/threonine kinase 3(Akt3)	-0.764
21- to 104-week	ENSRNOG00000021569	T-cell lymphoma invasion and metastasis 1(Tiam1)	-0.750
21- to 104-week	ENSRNOG00000022141	CTD small phosphatase like 2(Ctdspl2)	-0.750

Age	Ensembl ID	Gene name	ρ
21- to 104-week	ENSRNOG00000022156	coiled-coil domain containing 178(Ccdc178)	-0.750
21- to 104-week	ENSRNOG00000022162	PBX homeobox 3(Pbx3)	-0.639
21- to 104-week	ENSRNOG00000022249	myeloid/lymphoid or mixed-lineage leukemia; translocated to, 10(Mllt10)	-0.639
21- to 104-week	ENSRNOG00000022343	ALMS1, centrosome and basal body associated protein(Alms1)	-0.873
21- to 104-week	ENSRNOG00000022445	IQ motif containing F3(Iqcf3)	-0.750
21- to 104-week	ENSRNOG00000022720	ubiquitin specific peptidase 37(Usp37)	-0.639
21- to 104-week	ENSRNOG00000022845	centrosomal protein 70(Cep70)	-0.750
21- to 104-week	ENSRNOG00000023202	ubiquitin specific peptidase 15(Usp15)	-0.894
21- to 104-week	ENSRNOG00000023566	von Willebrand factor A domain containing 3B(Vwa3b)	-0.930
21- to 104-week	ENSRNOG00000023698	testis expressed 14, intercellular bridge forming factor(Tex14)	-0.750
21- to 104-week	ENSRNOG00000023807	cyclin Y-like 1(Ccnyl1)	-0.639
21- to 104-week	ENSRNOG00000023941	family with sequence similarity 178, member B(Fam178b)	-0.930
21- to 104-week	ENSRNOG00000024025	enhancer of mRNA decapping 4(Edc4)	-0.750
21- to 104-week	ENSRNOG00000024402	focadhesin(Focad)	-0.894
21- to 104-week	ENSRNOG00000024503	neuroblastoma amplified sequence(Nbas)	-0.894
21- to 104-week	ENSRNOG00000024521	spermatogenesis associated 16(Spata16)	-0.750
21- to 104-week	ENSRNOG00000024530	LYR motif containing 1(Lyrm1)	-0.930
21- to 104-week	ENSRNOG00000024545	coiled-coil domain containing 18(Ccdc18)	-0.639
21- to 104-week	ENSRNOG00000024563	glucosidase, alpha; neutral C(Ganc)	-0.930
21- to 104-week	ENSRNOG00000024808	serine threonine kinase 39(Stk39)	-0.873
21- to 104-week	ENSRNOG00000024837	transmembrane phosphoinositide 3-phosphatase and tensin homolog 2(Tpte2)	-0.930
21- to 104-week	ENSRNOG00000024870	ankyrin repeat and sterile alpha motif domain containing 1B(Anks1b)	-0.639
21- to 104-week	ENSRNOG00000024878	RNA binding motif protein 44(Rbm44)	-0.930
21- to 104-week	ENSRNOG00000025141	kizuna centrosomal protein(Kiz)	-0.894
21- to 104-week	ENSRNOG00000025332	CD109 molecule(Cd109)	-0.750
21- to 104-week	ENSRNOG00000025418	armadillo repeat containing 9(Armc9)	-0.894
21- to 104-week	ENSRNOG00000025420	CKLF-like MARVEL transmembrane domain containing 2A(Cmtm2a)	-0.750
21- to 104-week	ENSRNOG00000025528	EFR3 homolog A(Efr3a)	-0.750
21- to 104-week	ENSRNOG00000025551	regulator of G-protein signaling 22(Rgs22)	-0.930
21- to 104-week	ENSRNOG00000025672	testis expressed 11(Tex11)	-0.750
21- to 104-week	ENSRNOG00000025728	ADAM metallopeptidase domain 32(Adam32)	-0.930
21- to 104-week	ENSRNOG00000025787	sperm associated antigen 6(Spag6)	-0.930
21- to 104-week	ENSRNOG00000025823	RNA binding motif protein 46(Rbm46)	-0.894
21- to 104-week	ENSRNOG00000026180	leucine zipper and EF-hand containing transmembrane protein 2(Letm2)	-0.750
21- to 104-week	ENSRNOG00000026277	zinc finger CCCH type containing 6(Zc3h6)	-0.750
21- to 104-week	ENSRNOG00000026589	dpy-19 like 1(Dpy19l1)	-0.930
21- to 104-week	ENSRNOG00000026610	akirin 1(Akirin1)	-0.750
21- to 104-week	ENSRNOG00000026845	family with sequence similarity 81, member B(Fam81b)	-0.750
21- to 104-week	ENSRNOG00000027012	ubiquitin specific peptidase 54(Usp54)	-0.894
21- to 104-week	ENSRNOG00000027250	RNA binding motif protein 27(Rbm27)	-0.655

Age	Ensembl ID	Gene name	ρ
21- to 104-week	ENSRNOG00000027617	tetratricopeptide repeat domain 26(Ttc26)	-0.930
21- to 104-week	ENSRNOG00000027801	zona pellucida binding protein(Zpbp)	-0.878
21- to 104-week	ENSRNOG00000027929	DNA polymerase nu(Poln)	-0.894
21- to 104-week	ENSRNOG00000027992	DS cell adhesion molecule(Dscam)	-0.750
21- to 104-week	ENSRNOG00000028227	polybromo 1(Pbrm1)	-0.930
21- to 104-week	ENSRNOG00000028501	zinc finger CCCH-type containing 18(Zc3h18)	-0.750
21- to 104-week	ENSRNOG00000029235	Scm-like with four mbt domains 2(Sfmbt2)	-0.750
21- to 104-week	ENSRNOG00000029773	ATM serine/threonine kinase(Atm)	-0.930
21- to 104-week	ENSRNOG00000030705	cleavage and polyadenylation specific factor 1(Cpsf1)	-0.750
21- to 104-week	ENSRNOG00000031247	leucine, glutamate and lysine rich 1(Lekr1)	-0.894
21- to 104-week	ENSRNOG00000031479	high density lipoprotein binding protein(Hdlbp)	-0.750
21- to 104-week	ENSRNOG00000032180	coiled-coil domain containing 15(Ccdc15)	-0.639
21- to 104-week	ENSRNOG00000032570	structural maintenance of chromosomes 1B(Smc1b)	-0.750
21- to 104-week	ENSRNOG00000032574	scribbled planar cell polarity protein(Scrib)	-0.930
21- to 104-week	ENSRNOG00000033169	cytoplasmic polyadenylation element binding protein 4(Cpeb4)	-0.750
21- to 104-week	ENSRNOG00000033528	tolloid-like 1(Tll1)	-0.930
21- to 104-week	ENSRNOG00000034026	lysocardiolipin acyltransferase 1(Lclat1)	-0.750
21- to 104-week	ENSRNOG00000034272	protein inhibitor of activated STAT, 1(Pias1)	-0.639
21- to 104-week	ENSRNOG00000036601	exocyst complex component 6(Exoc6)	-0.750
21- to 104-week	ENSRNOG00000036675	BTB domain containing 16(Btbd16)	-0.639
21- to 104-week	ENSRNOG00000036964	Ral GTPase activating protein catalytic alpha subunit 2(Ralgapa2)	-0.659
21- to 104-week	ENSRNOG00000036980	zinc finger, GRF-type containing 1(Zgrf1)	-0.750
21- to 104-week	ENSRNOG00000037865	HORMA domain containing 2(Hormad2)	-0.894
21- to 104-week	ENSRNOG00000038002	coiled-coil domain containing 62(Ccdc62)	-0.878
21- to 104-week	ENSRNOG00000038242	similar to T-cell activation Rho GTPase-activating protein isoform b(LOC365791)	-0.930
21- to 104-week	ENSRNOG00000038436	similar to RIKEN cDNA D630029K19(RGD1307100)	-0.930
21- to 104-week	ENSRNOG00000039466	hyaluronoglucosaminidase 5(Hyal5)	-0.750
21- to 104-week	ENSRNOG00000039656	ATP-binding cassette, subfamily A (ABC1), member 17(Abca17)	-0.750
21- to 104-week	ENSRNOG00000042068	OTU deubiquitinase 7B(Otud7b)	-0.930
21- to 104-week	ENSRNOG00000042195	poly(A) binding protein, nuclear 1(Pabpn1)	-0.750
21- to 104-week	ENSRNOG00000042333	dynein, axonemal, light chain 1(Dnal1)	-0.639
21- to 104-week	ENSRNOG00000042359	phosphatidylinositol glycan anchor biosynthesis, class K(Pigk)	-0.750
21- to 104-week	ENSRNOG00000042432	NIMA-related kinase 11(Nek11)	-0.930
21- to 104-week	ENSRNOG00000042932	tetratricopeptide repeat domain 27(Ttc27)	-0.659
21- to 104-week	ENSRNOG00000043035	F-box and leucine-rich repeat protein 13(Fbxl13)	-0.639
21- to 104-week	ENSRNOG00000045585	dpy-19 like 2(Dpy19l2)	-0.894
21- to 104-week	ENSRNOG00000045639	similar to KIAA0825 protein(RGD1560883)	-0.750
21- to 104-week	ENSRNOG00000046023	exocyst complex component 4(Exoc4)	-0.930
21- to 104-week	ENSRNOG00000048411	ubiquitin-like with PHD and ring finger domains 1(Uhrf1)	-0.750
21- to 104-week	ENSRNOG00000048719	dystrobrevin binding protein 1(Dtnbp1)	-0.930
21- to 104-week	ENSRNOG00000049507	septin 10(Sept10)	-0.894

Age	Ensembl ID	Gene name	*ρ*
21- to 104-week	ENSRNOG00000050191	coiled-coil domain containing 7(Ccdc7)	-0.894
21- to 104-week	ENSRNOG00000050233	coiled-coil alpha-helical rod protein 1(Cchcr1)	-0.750
21- to 104-week	ENSRNOG00000050317	UHRF1 binding protein 1-like(Uhrf1bp1l)	-0.750
21- to 104-week	ENSRNOG00000052688	dynein, axonemal, heavy chain 2(Dnah2)	-0.750
21- to 104-week	ENSRNOG00000053060	zinc finger, C3H1-type containing(Zfc3h1)	-0.639
21- to 104-week	ENSRNOG00000054058	oxysterol binding protein-like 1A(Osbpl1a)	-0.750
21- to 104-week	ENSRNOG00000054331	WD repeat containing planar cell polarity effector(Wdpcp)	-0.878
21- to 104-week	ENSRNOG00000054475	uncharacterized LOC102550841(LOC102550841)	-0.750
21- to 104-week	ENSRNOG00000055103	cystic fibrosis transmembrane conductance regulator(Cftr)	-0.750
21- to 104-week	ENSRNOG00000055790	nucleoporin 210-like(Nup210l)	-0.750
21- to 104-week	ENSRNOG00000056330	cyclic nucleotide binding domain containing 1(Cnbd1)	-0.750
21- to 104-week	ENSRNOG00000056654	dyslexia susceptibility 1 candidate 1(Dyx1c1)	-0.750
21- to 104-week	ENSRNOG00000057102	platelet-activating factor acetylhydrolase 1b, catalytic subunit 2(Pafah1b2)	-0.750
21- to 104-week	ENSRNOG00000057315	potassium voltage-gated channel subfamily H member 3(Kcnh3)	-0.894
21- to 104-week	ENSRNOG00000057372	methyltransferase like 6(Mettl6)	-0.878
21- to 104-week	ENSRNOG00000057378	C-type lectin domain family 16, member A(Clec16a)	-0.930
21- to 104-week	ENSRNOG00000057740	coiled-coil domain containing 148(Ccdc148)	-0.930
21- to 104-week	ENSRNOG00000057815	coiled-coil domain containing 191(Ccdc191)	-0.878
21- to 104-week	ENSRNOG00000057945	Fanconi anemia, complementation group G(Fancg)	-0.930
21- to 104-week	ENSRNOG00000058275	sperm flagellar 2(Spef2)	-0.750
21- to 104-week	ENSRNOG00000058681	testis specific 10(Tsga10)	-0.750
21- to 104-week	ENSRNOG00000058733	ArfGAP with SH3 domain, ankyrin repeat and PH domain 1(Asap1)	-0.750
21- to 104-week	ENSRNOG00000059091	similar to ankyrin-like protein(RGD1563748)	-0.750
21- to 104-week	ENSRNOG00000059276	similar to hypothetical protein FLJ10706(LOC498265)	-0.930
21- to 104-week	ENSRNOG00000059659	zinc finger AN1-type containing 3(Zfand3)	-0.750
21- to 104-week	ENSRNOG00000059865	dynein, axonemal, heavy chain 12(Dnah12)	-0.783
21- to 104-week	ENSRNOG00000060100	kinetochore scaffold 1(Knl1)	-0.639
21- to 104-week	ENSRNOG00000060367	regulatory factor X, 7(Rfx7)	-0.659
21- to 104-week	ENSRNOG00000060479	Sp3 transcription factor(Sp3)	-0.750
21- to 104-week	ENSRNOG00000060561	glycerophosphodiester phosphodiesterase domain containing 1(Gdpd1)	-0.894
21- to 104-week	ENSRNOG00000060626	DNA polymerase kappa(Polk)	-0.639
21- to 104-week	ENSRNOG00000060984	dynein, axonemal, heavy chain 7(Dnah7)	-0.930
21- to 104-week	ENSRNOG00000061040	HECT and RLD domain containing E3 ubiquitin protein ligase 4(Herc4)	-0.873
21- to 104-week	ENSRNOG00000061050	membrane bound O-acyltransferase domain containing 2(Mboat2)	-0.639
21- to 104-week	ENSRNOG00000061543	adaptor-related protein complex 2, beta 1 subunit(Ap2b1)	-0.750
21- to 104-week	ENSRNOG00000061795	RNA binding motif protein 4B(Rbm4b)	-0.750

Note – The ρ values were calculated with *Spearman*'s rank correlation test. Only the circRNAs with $|\rho| > 0.6$ were listed.

Supplementary Table S3. The host genes of the age-dependent circRNAs in adrenal gland, heart, kidney, liver, lung, muscle, spleen, thymus, and uterus

Tissue	Ensembl ID	Gene name	*ρ*
Adrenal	ENSRNOG00000002983	nuclear factor 1 X(Nf1x)	0.842
Adrenal	ENSRNOG00000006997	amyloid beta precursor protein(App)	0.655
Adrenal	ENSRNOG00000008709	Rho GTPase activating protein 32(Arhgap32)	0.666
Adrenal	ENSRNOG00000010853	cholinergic receptor nicotinic alpha 7 subunit(Chrna7)	0.785
Adrenal	ENSRNOG00000020769	CREB3 regulatory factor(Crebrf)	0.639
Adrenal	ENSRNOG00000029614	roundabout guidance receptor 1(Robo1)	0.647
Adrenal	ENSRNOG00000032215	chromodomain Y-like(Cdyl)	0.733
Heart	ENSRNOG00000001469	elastin(Eln)	-0.808
Heart	ENSRNOG00000003357	collagen type III alpha 1 chain(Col3a1)	-0.807
Heart	ENSRNOG00000003669	myocardin(Myocd)	-0.638
Heart	ENSRNOG00000004444	IKAROS family zinc finger 1(Ikzf1)	0.675
Heart	ENSRNOG00000015519	carboxylesterase 1D(Ces1d)	0.844
Heart	ENSRNOG00000017583	mitogen-activated protein kinase associated protein 1(Mapkap1)	-0.634
Heart	ENSRNOG00000017879	GRB2-associated binding protein 1(Gab1)	-0.624
Heart	ENSRNOG00000023679	PR/SET domain 5(Prdm5)	-0.718
Heart	ENSRNOG00000050183	RT1 class I, locus1(RT1-CE1)	0.624
Kidney	ENSRNOG00000011504	A-kinase anchoring protein 2(Akap2)	0.787
Kidney	ENSRNOG00000011517	TNF receptor superfamily member 21(Tnfrsf21)	0.615
Kidney	ENSRNOG00000013328	RNA binding protein with multiple splicing(Rbpms)	0.632
Kidney	ENSRNOG00000017172	multivesicular body subunit 12B(Mvb12b)	-0.607
Kidney	ENSRNOG00000037851	scaffolding protein involved in DNA repair(Spidr)	0.793
Lung	ENSRNOG00000000142	plexin domain containing 2(Plxdc2)	-0.652
Lung	ENSRNOG00000000277	tet methylcytosine dioxygenase 1(Tet1)	-0.716
Lung	ENSRNOG00000001469	elastin(Eln)	-0.798
Lung	ENSRNOG00000003357	collagen type III alpha 1 chain(Col3a1)	-0.772
Lung	ENSRNOG00000004208	cysteine rich transmembrane BMP regulator 1(Crim1)	0.651
Lung	ENSRNOG00000004444	IKAROS family zinc finger 1(Ikzf1)	0.817
Lung	ENSRNOG00000011063	DENN domain containing 1B(Dennd1b)	0.680
Lung	ENSRNOG00000011105	ADP-ribosylation factor like GTPase 15(Arl15)	-0.754
Lung	ENSRNOG00000014647	core-binding factor, beta subunit(Cbfb)	0.662
Lung	ENSRNOG00000015519	carboxylesterase 1D(Ces1d)	0.633
Lung	ENSRNOG00000017879	GRB2-associated binding protein 1(Gab1)	-0.606
Lung	ENSRNOG00000019689	von Willebrand factor(Vwf)	0.685
Lung	ENSRNOG00000032247	B-cell scaffold protein with ankyrin repeats 1(Bank1)	0.675
Lung	ENSRNOG00000037080	ADAM metallopeptidase with thrombospondin type 1 motif, 17(Adamts17)	-0.819
Lung	ENSRNOG00000045605	UDP-glucuronate decarboxylase 1(Uxs1)	0.720
Lung	ENSRNOG00000050183	RT1 class I, locus1(RT1-CE1)	0.754
Liver	ENSRNOG00000016213	single-stranded DNA binding protein 2(Ssbp2)	-0.607
Liver	ENSRNOG00000021405	cytochrome P450, family 2, subfamily c, polypeptide 7(Cyp2c7)	0.779

Tissue	Ensembl ID	Gene name	ρ
Liver	ENSRNOG00000034013	acetyl-CoA carboxylase alpha(Acaca)	0.720
Muscle	ENSRNOG00000001120	mediator complex subunit 13-like(Med13l)	-0.620
Muscle	ENSRNOG00000001682	tetratricopeptide repeat domain 3(Ttc3)	-0.638
Muscle	ENSRNOG00000002214	kelch-like family member 8(Klhl8)	0.713
Muscle	ENSRNOG00000002451	fibronectin type III domain containing 3C2(Fnd3c2)	-0.775
Muscle	ENSRNOG00000003357	collagen type III alpha 1 chain(Col3a1)	-0.832
Muscle	ENSRNOG00000004050	SNF related kinase(Snrk)	-0.697
Muscle	ENSRNOG00000011504	A-kinase anchoring protein 2(Akap2)	-0.668
Muscle	ENSRNOG00000016213	single-stranded DNA binding protein 2(Ssbp2)	0.802
Muscle	ENSRNOG00000016684	WNK lysine deficient protein kinase 2(Wnk2)	0.602
Muscle	ENSRNOG00000023679	PR/SET domain 5(Prdm5)	-0.768
Muscle	ENSRNOG00000025539	vacuolar protein sorting 13A(Vps13a)	0.631
Muscle	ENSRNOG00000058068	obscurin, cytoskeletal calmodulin and titin-interacting RhoGEF(Obscn)	0.672
Spleen	ENSRNOG00000011278	serine/threonine kinase 3(Stk3)	0.654
Spleen	ENSRNOG00000013368	phospholipase C-like 2(Plcl2)	-0.625
Spleen	ENSRNOG00000045605	UDP-glucuronate decarboxylase 1(Uxs1)	0.684
Thymus	ENSRNOG00000000443	complement C4-like(LOC103689965)	0.769
Thymus	ENSRNOG00000002983	nuclear factor 1 X(Nf1x)	0.917
Thymus	ENSRNOG00000003296	deoxycytidine kinase(Dck)	-0.612
Thymus	ENSRNOG00000005391	phosphatidylinositol-3,4,5-trisphosphate-dependent Rac exchange factor 2(Prex2)	0.609
Thymus	ENSRNOG00000005776	B-cell CLL/lymphoma 11B(Bcl11b)	-0.606
Thymus	ENSRNOG00000010210	solute carrier family 7 member 11(Slc7a11)	-0.766
Thymus	ENSRNOG00000010827	polypyrimidine tract binding protein 2(Ptbp2)	-0.626
Thymus	ENSRNOG00000013598	maternal embryonic leucine zipper kinase(Melk)	-0.613
Thymus	ENSRNOG00000013615	DNA nucleotidylexotransferase(Dntt)	-0.603
Thymus	ENSRNOG00000013884	pleckstrin and Sec7 domain containing 3(Psd3)	0.829
Thymus	ENSRNOG00000015519	carboxylesterase 1D(Ces1d)	0.808
Thymus	ENSRNOG00000016924	ATP citrate lyase(Acly)	0.683
Thymus	ENSRNOG00000017803	amyloid beta precursor protein binding family B member 1 interacting protein(Apbb1ip)	-0.627
Thymus	ENSRNOG00000028422	required for meiotic nuclear division 5 homolog A(Rmnd5a)	-0.620
Thymus	ENSRNOG00000034013	acetyl-CoA carboxylase alpha(Acaca)	0.938
Thymus	ENSRNOG00000045636	fatty acid synthase(Fasn)	0.718
Thymus	ENSRNOG00000055631	zinc finger protein 280D(Zfp280d)	-0.627
Uterus	ENSRNOG00000000277	tet methylcytosine dioxygenase 1(Tet1)	-0.615
Uterus	ENSRNOG00000000942	PAN3 poly(A) specific ribonuclease subunit(Pan3)	0.800
Uterus	ENSRNOG00000002093	transforming growth factor beta receptor 3(Tgfbr3)	0.661
Uterus	ENSRNOG00000003357	collagen type III alpha 1 chain(Col3a1)	-0.667
Uterus	ENSRNOG00000004036	sorting nexin 12(Snx12)	0.781
Uterus	ENSRNOG00000004444	IKAROS family zinc finger 1(Ikzf1)	0.646
Uterus	ENSRNOG00000004571	similar to CDNA sequence BC052040(RGD1563680)	-0.760
Uterus	ENSRNOG00000005191	tripartite motif-containing 44(Trim44)	-0.640

Tissue	Ensembl ID	Gene name	ρ
Uterus	ENSRNOG00000005291	solute carrier family 38, member 1(Slc38a1)	0.704
Uterus	ENSRNOG00000005434	spectrin, beta, non-erythrocytic 1(Sptbn1)	0.640
Uterus	ENSRNOG00000006553	basonuclin 2(Bnc2)	0.714
Uterus	ENSRNOG00000006905	HECT domain E3 ubiquitin protein ligase 1(Hectd1)	0.647
Uterus	ENSRNOG00000007127	Bardet-Biedl syndrome 5(Bbs5)	0.759
Uterus	ENSRNOG00000008709	Rho GTPase activating protein 32(Arhgap32)	-0.764
Uterus	ENSRNOG00000009690	ring finger protein 219(Rnf219)	0.613
Uterus	ENSRNOG00000009836	RNA binding motif protein 26(Rbm26)	0.765
Uterus	ENSRNOG00000011584	G patch domain containing 1(Gpatch1)	0.640
Uterus	ENSRNOG00000012021	catenin, beta like 1(Ctnnbl1)	0.640
Uterus	ENSRNOG00000012271	CCR4-NOT transcription complex, subunit 1(Cnot1)	0.629
Uterus	ENSRNOG00000012698	chondroitin sulfate synthase 1(Chsy1)	0.706
Uterus	ENSRNOG00000013089	kinesin family member 13B(Kif13b)	0.708
Uterus	ENSRNOG00000013844	family with sequence similarity 172, member A(Fam172a)	0.629
Uterus	ENSRNOG00000013884	pleckstrin and Sec7 domain containing 3(Psd3)	-0.655
Uterus	ENSRNOG00000015813	ubiquitin protein ligase E3 component n-recognin 2(Ubr2)	0.693
Uterus	ENSRNOG00000015843	integrin alpha FG-GAP repeat containing 1(Itfg1)	0.661
Uterus	ENSRNOG00000017758	similar to NICE-3(LOC361985)	0.608
Uterus	ENSRNOG00000019005	phosphodiesterase 8A(Pde8a)	0.663
Uterus	ENSRNOG00000019090	chaperonin containing TCP1 subunit 3(Cct3)	0.634
Uterus	ENSRNOG00000019333	homeodomain interacting protein kinase 1(Hipk1)	0.640
Uterus	ENSRNOG00000019577	ADAM metallopeptidase with thrombospondin type 1 motif, 19(Adamts19)	-0.661
Uterus	ENSRNOG00000019948	PHD finger protein 20(Phf20)	-0.682
Uterus	ENSRNOG00000020848	coenzyme Q8B(Coq8b)	0.693
Uterus	ENSRNOG00000024482	trinucleotide repeat containing 18(Tnrc18)	0.613
Uterus	ENSRNOG00000027249	Fanconi anemia, complementation group L(Fancl)	0.640
Uterus	ENSRNOG00000037227	YES proto-oncogene 1, Src family tyrosine kinase(Yes1)	0.676
Uterus	ENSRNOG00000045605	UDP-glucuronate decarboxylase 1(Uxs1)	0.703
Uterus	ENSRNOG00000047014	homer scaffolding protein 1(Homer1)	0.611
Uterus	ENSRNOG00000054294	regulation of nuclear pre-mRNA domain containing 2(Rprd2)	0.629
Uterus	ENSRNOG00000054890	filamin A(Flna)	0.604
Uterus	ENSRNOG00000057880	myosin heavy chain 11(Myh11)	0.788

Note – The ρ values were calculated with *Spearman*'s rank correlation test. Only the circRNAs with $|\rho| > 0.6$ were listed.

Supplementary Table S4. The details of the 320 tissue samples in the rat BodyMap dataset.

Sample	Tissue	Age (Week)	Sex	Replicate	Total reads
Adr_F_002_1	Adrenal	2	F	1	32,893,912
Adr_F_002_2	Adrenal	2	F	2	34,216,259
Adr_F_002_3	Adrenal	2	F	3	40,353,070
Adr_F_002_4	Adrenal	2	F	4	38,142,330
Adr_F_006_1	Adrenal	6	F	1	42,169,397
Adr_F_006_2	Adrenal	6	F	2	41,031,429
Adr_F_006_3	Adrenal	6	F	3	42,537,053
Adr_F_006_4	Adrenal	6	F	4	40,723,528
Adr_F_021_1	Adrenal	21	F	1	56,640,235
Adr_F_021_2	Adrenal	21	F	2	63,167,739
Adr_F_021_3	Adrenal	21	F	3	33,336,402
Adr_F_021_4	Adrenal	21	F	4	49,973,385
Adr_F_104_1	Adrenal	104	F	1	50,959,286
Adr_F_104_2	Adrenal	104	F	2	39,499,844
Adr_F_104_3	Adrenal	104	F	3	30,406,326
Adr_F_104_4	Adrenal	104	F	4	44,512,172
Adr_M_002_1	Adrenal	2	M	1	43,217,205
Adr_M_002_2	Adrenal	2	M	2	34,086,099
Adr_M_002_3	Adrenal	2	M	3	54,623,694
Adr_M_002_4	Adrenal	2	M	4	58,382,411
Adr_M_006_1	Adrenal	6	M	1	47,795,886
Adr_M_006_2	Adrenal	6	M	2	39,095,817
Adr_M_006_3	Adrenal	6	M	3	58,990,226
Adr_M_006_4	Adrenal	6	M	4	58,471,533
Adr_M_021_1	Adrenal	21	M	1	38,592,942
Adr_M_021_2	Adrenal	21	M	2	42,966,993
Adr_M_021_3	Adrenal	21	M	3	53,013,485
Adr_M_021_4	Adrenal	21	M	4	40,308,635
Adr_M_104_1	Adrenal	104	M	1	34,273,669
Adr_M_104_2	Adrenal	104	M	2	57,005,782
Adr_M_104_3	Adrenal	104	M	3	28,975,806
Adr_M_104_4	Adrenal	104	M	4	33,413,724
Brn_F_002_1	Brain	2	F	1	40,243,776
Brn_F_002_2	Brain	2	F	2	55,894,697
Brn_F_002_3	Brain	2	F	3	52,132,882
Brn_F_002_4	Brain	2	F	4	46,304,026
Brn_F_006_1	Brain	6	F	1	36,770,720
Brn_F_006_2	Brain	6	F	2	40,981,364
Brn_F_006_3	Brain	6	F	3	42,839,368
Brn_F_006_4	Brain	6	F	4	45,933,093
Brn_F_021_1	Brain	21	F	1	30,816,305
Brn_F_021_2	Brain	21	F	2	56,085,643

Sample	Tissue	Age (Week)	Sex	Replicate	Total reads
Brn_F_021_3	Brain	21	F	3	37,425,957
Brn_F_021_4	Brain	21	F	4	30,959,602
Brn_F_104_1	Brain	104	F	1	56,057,901
Brn_F_104_2	Brain	104	F	2	47,681,218
Brn_F_104_3	Brain	104	F	3	65,666,976
Brn_F_104_4	Brain	104	F	4	66,613,288
Brn_M_002_1	Brain	2	M	1	29,965,640
Brn_M_002_2	Brain	2	M	2	51,323,751
Brn_M_002_3	Brain	2	M	3	57,541,039
Brn_M_002_4	Brain	2	M	4	33,250,363
Brn_M_006_1	Brain	6	M	1	45,953,040
Brn_M_006_2	Brain	6	M	2	56,030,679
Brn_M_006_3	Brain	6	M	3	35,332,949
Brn_M_006_4	Brain	6	M	4	35,757,661
Brn_M_021_1	Brain	21	M	1	46,611,957
Brn_M_021_2	Brain	21	M	2	33,650,904
Brn_M_021_3	Brain	21	M	3	39,897,816
Brn_M_021_4	Brain	21	M	4	34,318,172
Brn_M_104_1	Brain	104	M	1	45,048,861
Brn_M_104_2	Brain	104	M	2	36,847,461
Brn_M_104_3	Brain	104	M	3	34,192,975
Brn_M_104_4	Brain	104	M	4	40,326,338
Hrt_F_002_1	Heart	2	F	1	71,753,459
Hrt_F_002_2	Heart	2	F	2	34,698,255
Hrt_F_002_3	Heart	2	F	3	53,175,352
Hrt_F_002_4	Heart	2	F	4	41,243,912
Hrt_F_006_1	Heart	6	F	1	52,332,678
Hrt_F_006_2	Heart	6	F	2	42,882,028
Hrt_F_006_3	Heart	6	F	3	62,408,439
Hrt_F_006_4	Heart	6	F	4	51,750,887
Hrt_F_021_1	Heart	21	F	1	38,346,452
Hrt_F_021_2	Heart	21	F	2	45,833,002
Hrt_F_021_3	Heart	21	F	3	33,774,162
Hrt_F_021_4	Heart	21	F	4	49,682,374
Hrt_F_104_1	Heart	104	F	1	51,186,027
Hrt_F_104_2	Heart	104	F	2	36,461,517
Hrt_F_104_3	Heart	104	F	3	42,692,898
Hrt_F_104_4	Heart	104	F	4	43,540,195
Hrt_M_002_1	Heart	2	M	1	53,390,578
Hrt_M_002_2	Heart	2	M	2	60,430,937
Hrt_M_002_3	Heart	2	M	3	49,064,469
Hrt_M_002_4	Heart	2	M	4	51,566,182
Hrt_M_006_1	Heart	6	M	1	57,904,914

Sample	Tissue	Age (Week)	Sex	Replicate	Total reads
Hrt_M_006_2	Heart	6	M	2	36,396,595
Hrt_M_006_3	Heart	6	M	3	39,431,813
Hrt_M_006_4	Heart	6	M	4	48,413,209
Hrt_M_021_1	Heart	21	M	1	56,776,103
Hrt_M_021_2	Heart	21	M	2	39,049,562
Hrt_M_021_3	Heart	21	M	3	52,427,869
Hrt_M_021_4	Heart	21	M	4	57,888,428
Hrt_M_104_1	Heart	104	M	1	54,938,916
Hrt_M_104_2	Heart	104	M	2	40,866,129
Hrt_M_104_3	Heart	104	M	3	39,569,732
Hrt_M_104_4	Heart	104	M	4	51,692,814
Kdn_F_002_1	Kidney	2	F	1	53,808,204
Kdn_F_002_2	Kidney	2	F	2	33,119,938
Kdn_F_002_3	Kidney	2	F	3	41,484,642
Kdn_F_002_4	Kidney	2	F	4	51,461,396
Kdn_F_006_1	Kidney	6	F	1	39,090,168
Kdn_F_006_2	Kidney	6	F	2	43,344,456
Kdn_F_006_3	Kidney	6	F	3	44,729,763
Kdn_F_006_4	Kidney	6	F	4	46,056,348
Kdn_F_021_1	Kidney	21	F	1	56,071,180
Kdn_F_021_2	Kidney	21	F	2	46,382,154
Kdn_F_021_3	Kidney	21	F	3	43,488,475
Kdn_F_021_4	Kidney	21	F	4	48,089,114
Kdn_F_104_1	Kidney	104	F	1	42,324,958
Kdn_F_104_2	Kidney	104	F	2	39,783,536
Kdn_F_104_3	Kidney	104	F	3	53,702,805
Kdn_F_104_4	Kidney	104	F	4	46,123,420
Kdn_M_002_1	Kidney	2	M	1	48,304,048
Kdn_M_002_2	Kidney	2	M	2	43,849,613
Kdn_M_002_3	Kidney	2	M	3	48,712,708
Kdn_M_002_4	Kidney	2	M	4	50,088,874
Kdn_M_006_1	Kidney	6	M	1	41,358,648
Kdn_M_006_2	Kidney	6	M	2	28,757,350
Kdn_M_006_3	Kidney	6	M	3	60,615,179
Kdn_M_006_4	Kidney	6	M	4	41,410,588
Kdn_M_021_1	Kidney	21	M	1	53,656,853
Kdn_M_021_2	Kidney	21	M	2	44,502,793
Kdn_M_021_3	Kidney	21	M	3	40,315,290
Kdn_M_021_4	Kidney	21	M	4	29,696,035
Kdn_M_104_1	Kidney	104	M	1	56,835,940
Kdn_M_104_2	Kidney	104	M	2	97,864,638
Kdn_M_104_3	Kidney	104	M	3	81,728,379
Kdn_M_104_4	Kidney	104	M	4	42,669,039

Sample	Tissue	Age (Week)	Sex	Replicate	Total reads
Lng_F_002_1	Lung	2	F	1	41,533,777
Lng_F_002_2	Lung	2	F	2	48,734,095
Lng_F_002_3	Lung	2	F	3	59,169,326
Lng_F_002_4	Lung	2	F	4	45,759,427
Lng_F_006_1	Lung	6	F	1	53,581,814
Lng_F_006_2	Lung	6	F	2	48,929,259
Lng_F_006_3	Lung	6	F	3	33,453,734
Lng_F_006_4	Lung	6	F	4	45,199,683
Lng_F_021_1	Lung	21	F	1	29,182,773
Lng_F_021_2	Lung	21	F	2	43,787,060
Lng_F_021_3	Lung	21	F	3	47,325,414
Lng_F_021_4	Lung	21	F	4	35,174,628
Lng_F_104_1	Lung	104	F	1	56,676,755
Lng_F_104_2	Lung	104	F	2	41,016,566
Lng_F_104_3	Lung	104	F	3	49,746,103
Lng_F_104_4	Lung	104	F	4	34,166,377
Lng_M_002_1	Lung	2	M	1	54,965,678
Lng_M_002_2	Lung	2	M	2	58,298,002
Lng_M_002_3	Lung	2	M	3	47,729,582
Lng_M_002_4	Lung	2	M	4	58,186,350
Lng_M_006_1	Lung	6	M	1	39,525,551
Lng_M_006_2	Lung	6	M	2	31,598,157
Lng_M_006_3	Lung	6	M	3	39,539,858
Lng_M_006_4	Lung	6	M	4	49,071,000
Lng_M_021_1	Lung	21	M	1	52,187,172
Lng_M_021_2	Lung	21	M	2	34,437,880
Lng_M_021_3	Lung	21	M	3	32,943,175
Lng_M_021_4	Lung	21	M	4	28,550,513
Lng_M_104_1	Lung	104	M	1	55,493,841
Lng_M_104_2	Lung	104	M	2	37,826,341
Lng_M_104_3	Lung	104	M	3	47,621,755
Lng_M_104_4	Lung	104	M	4	50,924,647
Lvr_F_002_1	Liver	2	F	1	33,947,155
Lvr_F_002_2	Liver	2	F	2	25,725,181
Lvr_F_002_3	Liver	2	F	3	24,771,704
Lvr_F_002_4	Liver	2	F	4	43,551,385
Lvr_F_006_1	Liver	6	F	1	28,171,973
Lvr_F_006_2	Liver	6	F	2	33,376,474
Lvr_F_006_3	Liver	6	F	3	35,287,971
Lvr_F_006_4	Liver	6	F	4	30,726,120
Lvr_F_021_1	Liver	21	F	1	28,475,954
Lvr_F_021_2	Liver	21	F	2	26,196,053
Lvr_F_021_3	Liver	21	F	3	33,018,173

Sample	Tissue	Age (Week)	Sex	Replicate	Total reads
Lvr_F_021_4	Liver	21	F	4	30,023,883
Lvr_F_104_1	Liver	104	F	1	35,099,606
Lvr_F_104_2	Liver	104	F	2	32,578,204
Lvr_F_104_3	Liver	104	F	3	18,614,381
Lvr_F_104_4	Liver	104	F	4	16,172,219
Lvr_M_002_1	Liver	2	M	1	27,999,674
Lvr_M_002_2	Liver	2	M	2	22,925,861
Lvr_M_002_3	Liver	2	M	3	24,667,720
Lvr_M_002_4	Liver	2	M	4	23,233,275
Lvr_M_006_1	Liver	6	M	1	26,738,812
Lvr_M_006_2	Liver	6	M	2	28,827,872
Lvr_M_006_3	Liver	6	M	3	26,486,982
Lvr_M_006_4	Liver	6	M	4	27,752,424
Lvr_M_021_1	Liver	21	M	1	16,038,547
Lvr_M_021_2	Liver	21	M	2	30,524,885
Lvr_M_021_3	Liver	21	M	3	23,910,727
Lvr_M_021_4	Liver	21	M	4	29,952,853
Lvr_M_104_1	Liver	104	M	1	35,494,631
Lvr_M_104_2	Liver	104	M	2	35,267,042
Lvr_M_104_3	Liver	104	M	3	61,371,908
Lvr_M_104_4	Liver	104	M	4	26,310,530
Msc_F_002_1	Muscle	2	F	1	41,071,320
Msc_F_002_2	Muscle	2	F	2	37,413,515
Msc_F_002_3	Muscle	2	F	3	37,549,646
Msc_F_002_4	Muscle	2	F	4	35,734,639
Msc_F_006_1	Muscle	6	F	1	28,431,285
Msc_F_006_2	Muscle	6	F	2	38,390,341
Msc_F_006_3	Muscle	6	F	3	61,926,744
Msc_F_006_4	Muscle	6	F	4	33,288,649
Msc_F_021_1	Muscle	21	F	1	23,079,460
Msc_F_021_2	Muscle	21	F	2	38,154,547
Msc_F_021_3	Muscle	21	F	3	33,688,569
Msc_F_021_4	Muscle	21	F	4	40,196,665
Msc_F_104_1	Muscle	104	F	1	37,432,419
Msc_F_104_2	Muscle	104	F	2	26,640,934
Msc_F_104_3	Muscle	104	F	3	29,905,201
Msc_F_104_4	Muscle	104	F	4	30,607,466
Msc_M_002_1	Muscle	2	M	1	36,763,639
Msc_M_002_2	Muscle	2	M	2	36,003,073
Msc_M_002_3	Muscle	2	M	3	29,338,941
Msc_M_002_4	Muscle	2	M	4	31,964,949
Msc_M_006_1	Muscle	6	M	1	31,221,239
Msc_M_006_2	Muscle	6	M	2	28,417,024

Sample	Tissue	Age (Week)	Sex	Replicate	Total reads
Msc_M_006_3	Muscle	6	M	3	24,777,202
Msc_M_006_4	Muscle	6	M	4	29,533,569
Msc_M_021_1	Muscle	21	M	1	55,840,635
Msc_M_021_2	Muscle	21	M	2	17,605,900
Msc_M_021_3	Muscle	21	M	3	30,123,611
Msc_M_021_4	Muscle	21	M	4	38,741,769
Msc_M_104_1	Muscle	104	M	1	33,021,047
Msc_M_104_2	Muscle	104	M	2	36,019,219
Msc_M_104_3	Muscle	104	M	3	31,353,357
Msc_M_104_4	Muscle	104	M	4	33,944,508
Spl_F_002_1	Spleen	2	F	1	28,716,189
Spl_F_002_2	Spleen	2	F	2	36,351,486
Spl_F_002_3	Spleen	2	F	3	34,258,486
Spl_F_002_4	Spleen	2	F	4	51,789,784
Spl_F_006_1	Spleen	6	F	1	54,045,434
Spl_F_006_2	Spleen	6	F	2	34,309,675
Spl_F_006_3	Spleen	6	F	3	38,556,135
Spl_F_006_4	Spleen	6	F	4	30,184,705
Spl_F_021_1	Spleen	21	F	1	31,082,131
Spl_F_021_2	Spleen	21	F	2	34,503,656
Spl_F_021_3	Spleen	21	F	3	29,582,856
Spl_F_021_4	Spleen	21	F	4	41,256,125
Spl_F_104_1	Spleen	104	F	1	37,500,858
Spl_F_104_2	Spleen	104	F	2	32,488,876
Spl_F_104_3	Spleen	104	F	3	39,118,194
Spl_F_104_4	Spleen	104	F	4	54,073,009
Spl_M_002_1	Spleen	2	M	1	38,111,982
Spl_M_002_2	Spleen	2	M	2	36,314,374
Spl_M_002_3	Spleen	2	M	3	40,490,649
Spl_M_002_4	Spleen	2	M	4	43,238,620
Spl_M_006_1	Spleen	6	M	1	41,280,233
Spl_M_006_2	Spleen	6	M	2	30,639,331
Spl_M_006_3	Spleen	6	M	3	43,923,217
Spl_M_006_4	Spleen	6	M	4	38,245,067
Spl_M_021_1	Spleen	21	M	1	32,298,070
Spl_M_021_2	Spleen	21	M	2	31,581,261
Spl_M_021_3	Spleen	21	M	3	54,567,455
Spl_M_021_4	Spleen	21	M	4	52,645,185
Spl_M_104_1	Spleen	104	M	1	37,498,789
Spl_M_104_2	Spleen	104	M	2	42,657,126
Spl_M_104_3	Spleen	104	M	3	33,966,572
Spl_M_104_4	Spleen	104	M	4	40,754,322
Thm_F_002_1	Thymus	2	F	1	46,133,374

Sample	Tissue	Age (Week)	Sex	Replicate	Total reads
Thm_F_002_2	Thymus	2	F	2	36,796,576
Thm_F_002_3	Thymus	2	F	3	45,511,776
Thm_F_002_4	Thymus	2	F	4	31,002,656
Thm_F_006_1	Thymus	6	F	1	32,829,354
Thm_F_006_2	Thymus	6	F	2	39,658,155
Thm_F_006_3	Thymus	6	F	3	31,322,541
Thm_F_006_4	Thymus	6	F	4	33,327,997
Thm_F_021_1	Thymus	21	F	1	49,721,098
Thm_F_021_2	Thymus	21	F	2	36,924,211
Thm_F_021_3	Thymus	21	F	3	34,288,623
Thm_F_021_4	Thymus	21	F	4	35,435,648
Thm_F_104_1	Thymus	104	F	1	41,321,268
Thm_F_104_2	Thymus	104	F	2	31,468,357
Thm_F_104_3	Thymus	104	F	3	50,756,861
Thm_F_104_4	Thymus	104	F	4	50,994,147
Thm_M_002_1	Thymus	2	M	1	44,737,308
Thm_M_002_2	Thymus	2	M	2	39,654,834
Thm_M_002_3	Thymus	2	M	3	28,234,075
Thm_M_002_4	Thymus	2	M	4	40,710,541
Thm_M_006_1	Thymus	6	M	1	37,565,504
Thm_M_006_2	Thymus	6	M	2	50,467,811
Thm_M_006_3	Thymus	6	M	3	62,668,840
Thm_M_006_4	Thymus	6	M	4	40,138,219
Thm_M_021_1	Thymus	21	M	1	30,419,204
Thm_M_021_2	Thymus	21	M	2	28,449,225
Thm_M_021_3	Thymus	21	M	3	40,419,495
Thm_M_021_4	Thymus	21	M	4	35,969,269
Thm_M_104_1	Thymus	104	M	1	50,067,621
Thm_M_104_2	Thymus	104	M	2	46,400,550
Thm_M_104_3	Thymus	104	M	3	58,411,261
Thm_M_104_4	Thymus	104	M	4	30,266,828
Tst_M_002_1	Testes	2	M	1	38,786,378
Tst_M_002_2	Testes	2	M	2	46,510,409
Tst_M_002_3	Testes	2	M	3	55,939,035
Tst_M_002_4	Testes	2	M	4	40,535,357
Tst_M_006_1	Testes	6	M	1	32,451,684
Tst_M_006_2	Testes	6	M	2	34,489,765
Tst_M_006_3	Testes	6	M	3	64,845,426
Tst_M_006_4	Testes	6	M	4	45,280,342
Tst_M_021_1	Testes	21	M	1	44,266,539
Tst_M_021_2	Testes	21	M	2	43,885,804
Tst_M_021_3	Testes	21	M	3	53,823,609
Tst_M_021_4	Testes	21	M	4	48,442,971

Sample	Tissue	Age (Week)	Sex	Replicate	Total reads
Tst_M_104_1	Testes	104	M	1	39,935,182
Tst_M_104_2	Testes	104	M	2	37,170,564
Tst_M_104_3	Testes	104	M	3	44,451,678
Tst_M_104_4	Testes	104	M	4	52,471,109
Utr_F_002_1	Uterus	2	F	1	39,126,625
Utr_F_002_2	Uterus	2	F	2	33,956,066
Utr_F_002_3	Uterus	2	F	3	31,442,276
Utr_F_002_4	Uterus	2	F	4	42,833,061
Utr_F_006_1	Uterus	6	F	1	51,709,494
Utr_F_006_2	Uterus	6	F	2	48,933,982
Utr_F_006_3	Uterus	6	F	3	54,316,703
Utr_F_006_4	Uterus	6	F	4	36,371,273
Utr_F_021_1	Uterus	21	F	1	28,031,893
Utr_F_021_2	Uterus	21	F	2	56,435,414
Utr_F_021_3	Uterus	21	F	3	64,490,643
Utr_F_021_4	Uterus	21	F	4	49,674,463
Utr_F_104_1	Uterus	104	F	1	43,791,901
Utr_F_104_2	Uterus	104	F	2	42,885,150
Utr_F_104_3	Uterus	104	F	3	54,397,342
Utr_F_104_4	Uterus	104	F	4	45,878,141
