# Supplementary material for: Rat BodyMap transcriptomes reveal unique circular RNA features across tissue types and developmental stages
Source: RNA. 2018 Nov;24(11):1443–56. doi: 10.1261/rna.067132.118 (PMC6191709; doi:10.1261/rna.067132.118)
Supplement: Supplemental Material [file supp_067132.118_Supplemental_Figures.pdf]

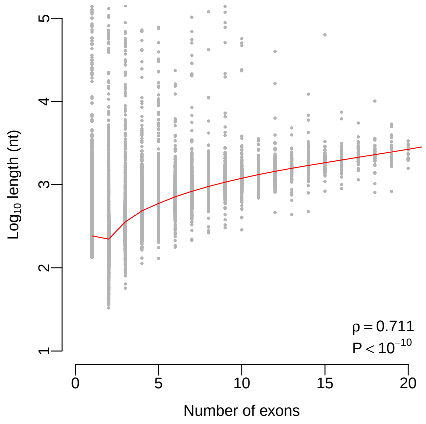

Supplementary Figure S1. Correlation between exon numbers and length of exonic circRNAs in rats. The red curve shows *Lowess* smoothed data. The correlation coefficient (ρ) and P-value were calculated with *Spearman*'s rank correlation test.

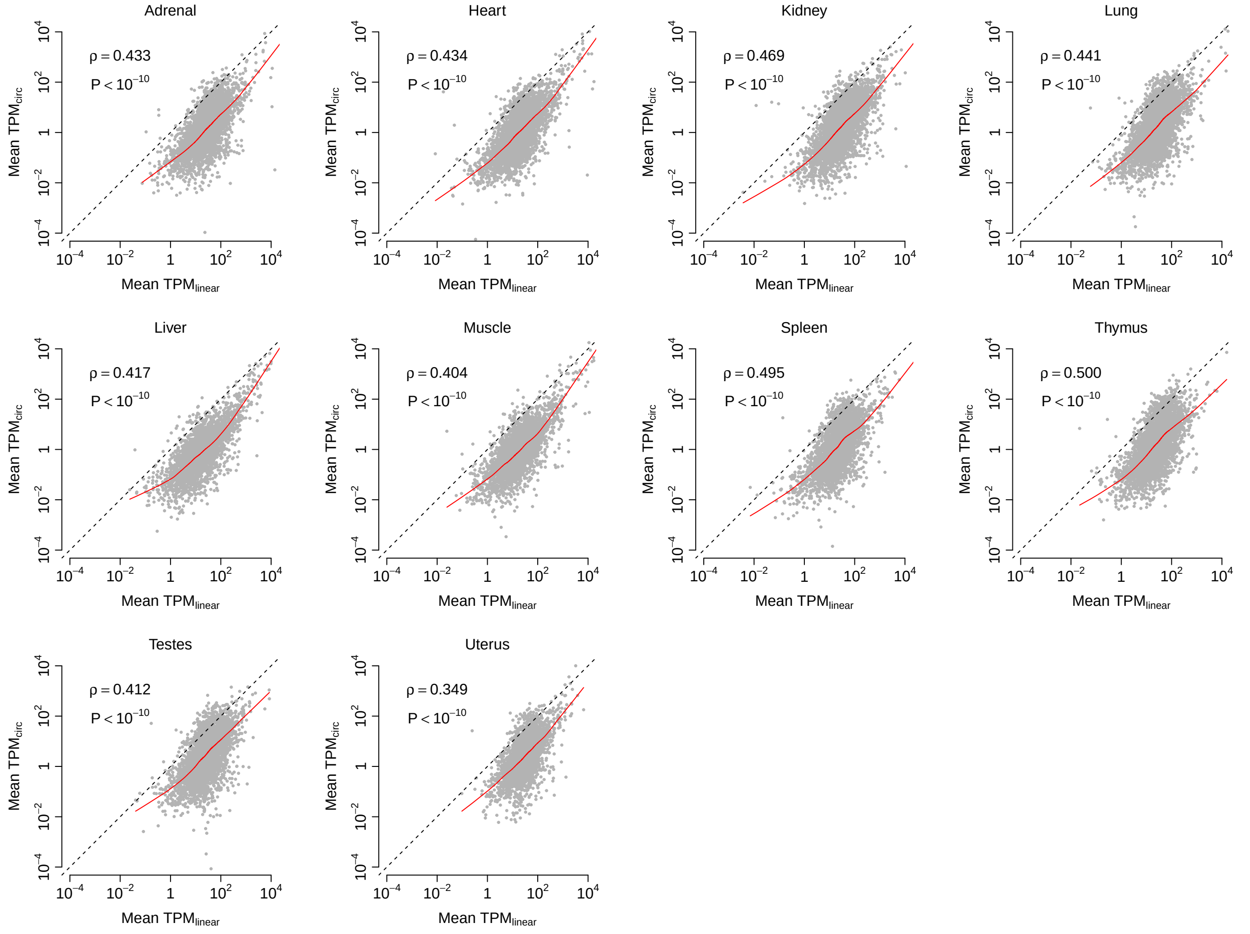

Supplementary Figure S2. Correlation between mean TPM_{linear} and mean TPM_{circ} values in rat tissues. Each dot represents one host gene. The dots above the diagonal denote the host genes with higher circRNA expression relative to their linear transcripts. The red curve represents *Lowess* smoothed data. The correlation coefficient (ρ) and P-value were calculated with *Spearman*'s rank correlation test.

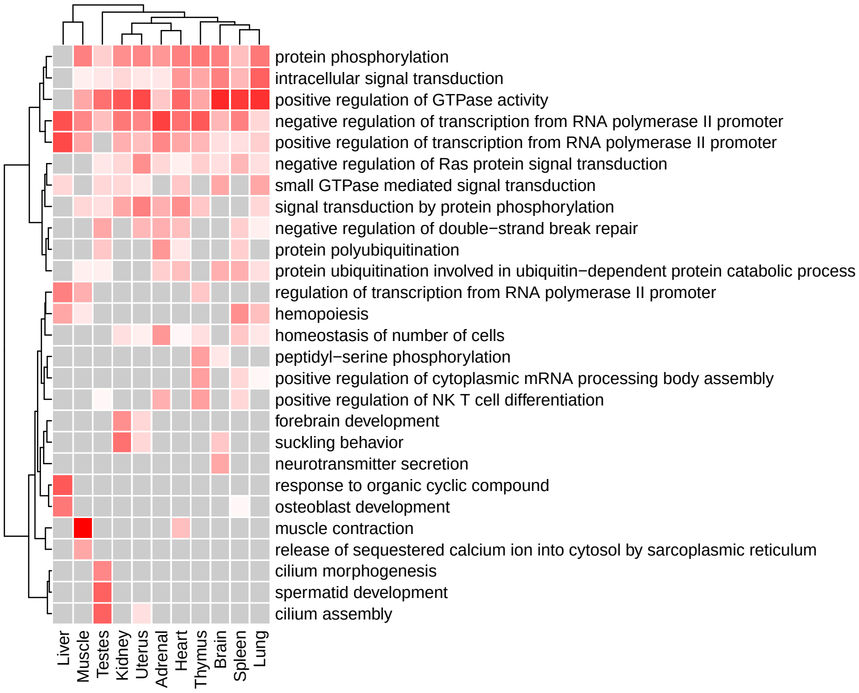

Supplementary Figure S3. The top GOBP terms associated with the circRNAs with relatively higher expression levels compared with their linear counterparts. For each tissue type, the top five GOBP terms were picked up. Because there were several common GOBP terms associated with these circRNAs across almost all the tissue types, in total 27 GOBP terms were listed. The association between the GOBP terms and these special circRNAs was measured based on the Z-score calculated from Fisher's exact test. Darker red indicates a stronger association, while lighter red indicates a weaker association. Grey indicates no association.

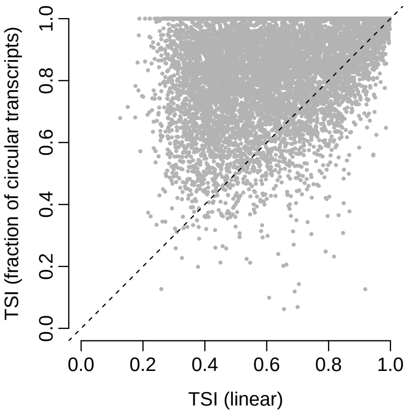

Supplementary Figure S4. Paired comparison of *TSI*. TSI was calculated based on linear RNA expression and fraction of circular transcripts, respectively. Each dot represents one host gene. The dots above the diagonal denote the host genes with a higher circRNA fraction based TSI compared with their linear counterparts.

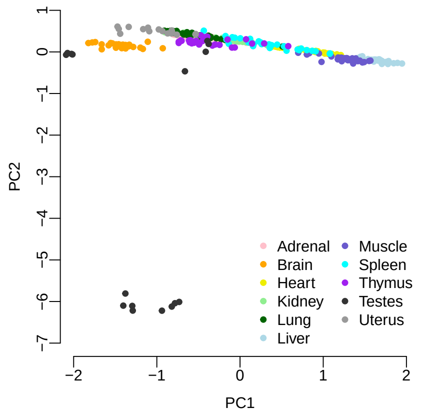

Supplementary Figure S5. PCA of mRNA expression. Each dot represents one tissue sample. PC1: the first principal component; PC2: the second principal component.

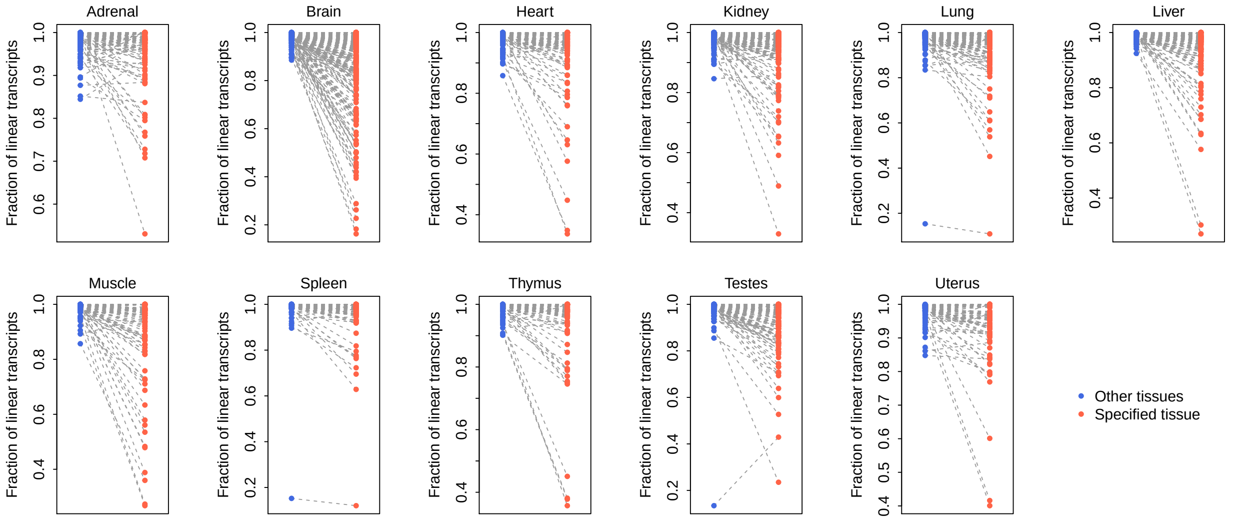

Supplementary Figure S6. Fraction of linear transcripts of tissue-specific mRNAs. The splicing ratio of tissue-specific mRNAs in the specific tissue was compared against those of all other tissue types. Each dot represents one tissue-specific mRNA. We observed a consistently lower fraction of linear transcripts for the tissue-specific mRNAs in the specific tissues (paired *Wilcoxon* test: P-value $< 10^{-10}$).

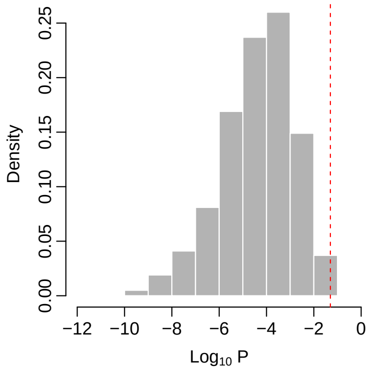

Supplementary Figure S7. Histogram of P-values computed by paired comparison of correlation coefficients (ρ) between the resampled circRNAs and their linear counterparts in rat brain. The ρ values were calculated between age and the expression of linear/circular transcripts using *Spearman*s rank correlation test. We generated 1,000 random circRNA sets by randomly picking up circRNAs expressed in rat brain. For each random circRNA set, the ρ values of the circRNAs were compared against the ρ values of their linear counterparts by one-tailed paired t-test. We found that $> 95\%$ of the P-values generated by t-test were less than 0.05 (the red dash line).

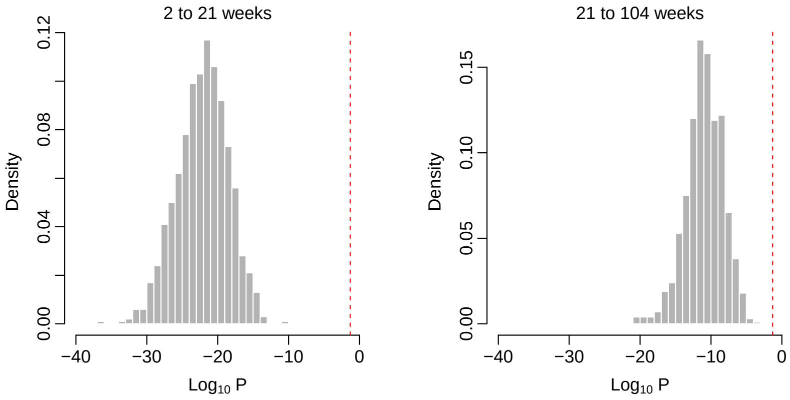

Supplementary Figure S8. Histogram of P-values computed by paired comparison of correlation coefficients (ρ) between the resampled circRNAs and their linear counterparts in rat testes. The ρ values were calculated between age and the expression of linear/circular transcripts using *Spearman*'s rank correlation test. We generated 1,000 random circRNA sets by randomly picking up circRNAs expressed in rat brain. For each random circRNA set, the ρ values of the circRNAs were compared against the ρ values of their linear counterparts by either right-tailed paired t-test (2 weeks to 21 weeks) or left-tailed paired t-test (21 weeks to 104 weeks). We found that all the P-values generated by t-test were less than 0.05 (the red dash line).
